# Near real-time multi-class segmentation for intravascular optical coherence tomography using knowledge distillation

**DOI:** 10.1093/ehjdh/ztag138

**Published:** 2026-09-02

**Authors:** Ruben G A van der Waerden, Rick H J A Volleberg, Pierandrea Cancian, Joske L van der Zande, Thijs J Luttikholt, Xiaojin Gu, Leah Heil, Jan-Quinten Mol, Kensuke Nishimiya, Tomasz Roleder, Clara I Sánchez, Bram van Ginneken, Jos Thannhauser, Niels van Royen, Ivana Išgum, Simone Saitta

**Affiliations:** Department of Cardiology, Radboud University Medical Center, PO Box 9101, 6500 HB, Nijmegen, the Netherlands; Diagnostic Image Analysis Group, Radboud University Medical Center, Geert Grooteplein Zuid 10, 6525 GA, Nijmegen, the Netherlands; Department of Cardiology, Radboud University Medical Center, PO Box 9101, 6500 HB, Nijmegen, the Netherlands; Department of Biomedical Engineering and Physics, Amsterdam University Medical Center, Amsterdam, the Netherlands; Informatics Institute, University of Amsterdam, Amsterdam, the Netherlands; Amsterdam Cardiovascular Sciences, Amsterdam UMC, Amsterdam, the Netherlands; Department of Cardiology, Radboud University Medical Center, PO Box 9101, 6500 HB, Nijmegen, the Netherlands; Diagnostic Image Analysis Group, Radboud University Medical Center, Geert Grooteplein Zuid 10, 6525 GA, Nijmegen, the Netherlands; Department of Cardiology, Radboud University Medical Center, PO Box 9101, 6500 HB, Nijmegen, the Netherlands; Diagnostic Image Analysis Group, Radboud University Medical Center, Geert Grooteplein Zuid 10, 6525 GA, Nijmegen, the Netherlands; Department of Biomedical Engineering and Physics, Amsterdam University Medical Center, Amsterdam, the Netherlands; Informatics Institute, University of Amsterdam, Amsterdam, the Netherlands; Amsterdam Cardiovascular Sciences, Amsterdam UMC, Amsterdam, the Netherlands; Department of Cardiology, Radboud University Medical Center, PO Box 9101, 6500 HB, Nijmegen, the Netherlands; Diagnostic Image Analysis Group, Radboud University Medical Center, Geert Grooteplein Zuid 10, 6525 GA, Nijmegen, the Netherlands; Department of Cardiology, Radboud University Medical Center, PO Box 9101, 6500 HB, Nijmegen, the Netherlands; Department of Cardiovascular Medicine, Tohoku University Graduate School of Medicine, Sendai, Japan; Faculty of Medicine, Wrocław University of Science and Technology, Wrocław, Poland; Department of Biomedical Engineering and Physics, Amsterdam University Medical Center, Amsterdam, the Netherlands; Informatics Institute, University of Amsterdam, Amsterdam, the Netherlands; Diagnostic Image Analysis Group, Radboud University Medical Center, Geert Grooteplein Zuid 10, 6525 GA, Nijmegen, the Netherlands; Department of Cardiology, Radboud University Medical Center, PO Box 9101, 6500 HB, Nijmegen, the Netherlands; Diagnostic Image Analysis Group, Radboud University Medical Center, Geert Grooteplein Zuid 10, 6525 GA, Nijmegen, the Netherlands; Department of Cardiology, Radboud University Medical Center, PO Box 9101, 6500 HB, Nijmegen, the Netherlands; Department of Biomedical Engineering and Physics, Amsterdam University Medical Center, Amsterdam, the Netherlands; Informatics Institute, University of Amsterdam, Amsterdam, the Netherlands; Department of Radiology and Nuclear Medicine, Amsterdam University Medical Center University of Amsterdam, Amsterdam, the Netherlands; Department of Radiology, Mayo Clinic, Rochester, MN, USA; Department of Biomedical Engineering and Physics, Amsterdam University Medical Center, Amsterdam, the Netherlands; Informatics Institute, University of Amsterdam, Amsterdam, the Netherlands; Amsterdam Cardiovascular Sciences, Amsterdam UMC, Amsterdam, the Netherlands

**Keywords:** Optical coherence tomography, Knowledge distillation, Coronary intervention, Deep learning, Cardiovascular imaging

## Abstract

**Aims:**

Intravascular optical coherence tomography (OCT) enables high-resolution imaging of the coronary vessel wall, but manual image interpretation is time-consuming and existing automated approaches often require high computational resources and exhibit slow inference times, limiting clinical use. We developed OCT-AID-lite, a neural network for near-real-time multi-class OCT segmentation leveraging knowledge distillation and semi-supervised learning to accelerate inference while maintaining segmentation accuracy.

**Methods and results:**

A state-of-the-art model (OCT-AID) guided a compact U-Net–based student model (OCT-AID-lite) through knowledge distillation-based supervision. OCT-AID-lite was trained on 3466 manually annotated and 137 961 pseudo-labelled frames after automated quality control. On 389 internal test frames, OCT-AID-lite achieved a forward-pass time of 0.10 s for a 540-frame pullback, compared with 24.22 s for the OCT-AID model (*P*  *<*  *0.01*). Including pre- and post-processing, total processing time was 5.50 s for OCT-AID-lite, compared with 30.62 s for OCT-AID (*P*  *<*  *0.01*). For lipid and calcium plaque classification, OCT-AID-lite reached sensitivity/specificity of 98.1%/74.4% and 89.5%/87.5%, respectively. Pixel-wise segmentation performance on true-positive frames was high for guidewire, catheter, lumen, intima, and media (Dice: 0.79–0.99), moderate to high for sidebranch, lipid, and calcium (Dice: 0.76–0.78), and more variable for rare complex classes (Dice: 0.39–0.67). On an independent external test set, model predictions were in agreement with expert assessment.

**Conclusion:**

OCT-AID-lite enables accurate OCT segmentation in near real-time, allowing efficient quantitative characterization of plaque and vessel structures.

## Graphical Abstract

**Graphical Abstract:**
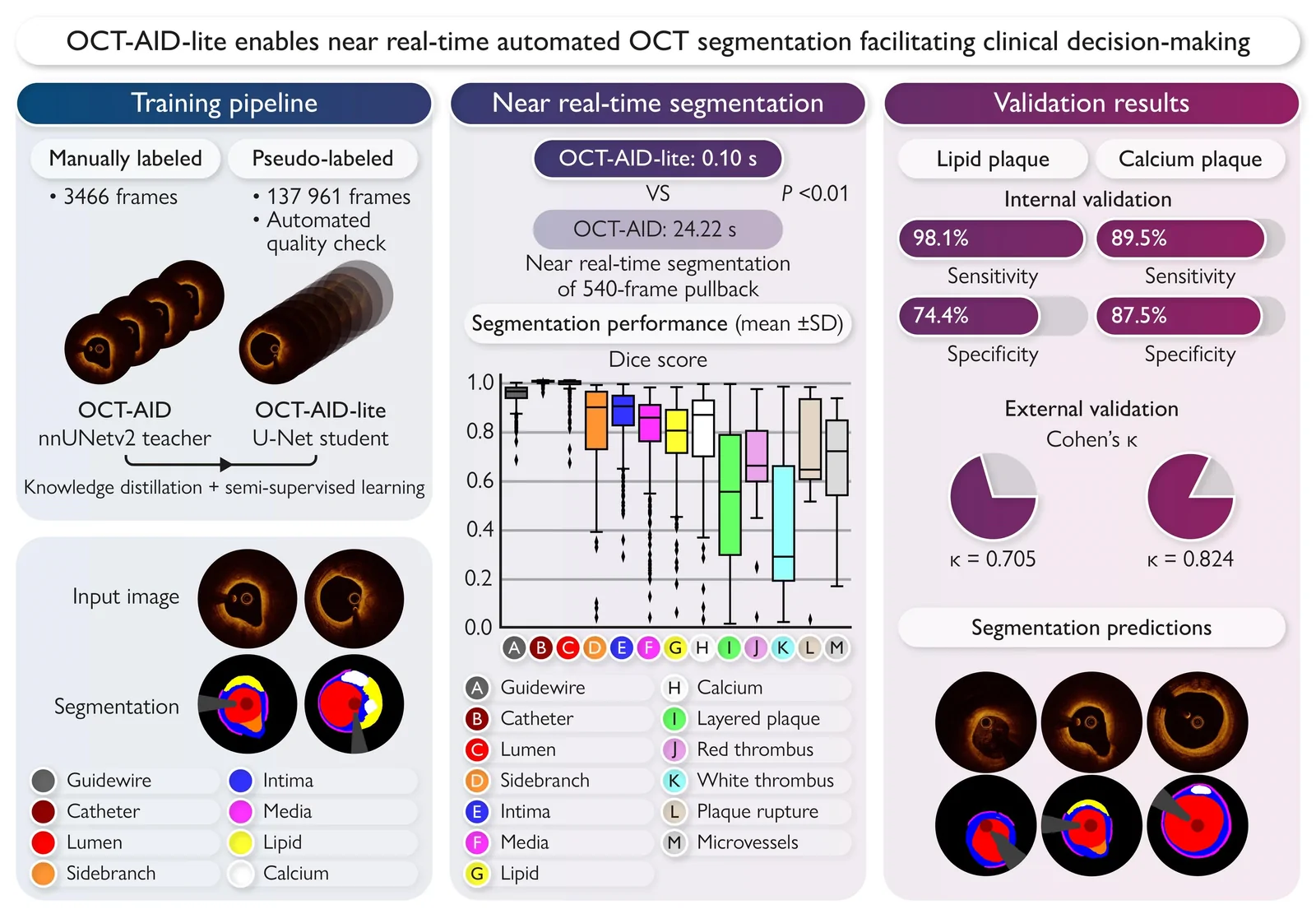

## Introduction

Accurate evaluation of coronary lesions is essential during percutaneous coronary intervention (PCI). Although coronary angiography remains the clinical reference standard for assessing luminal stenosis, it provides limited information on plaque composition.^1^ Intravascular optical coherence tomography (OCT) offers high-resolution imaging of the vessel wall, enabling detailed characterization of plaque morphology. Its expanding role in PCI guidance is acknowledged in contemporary clinical guidelines,^2,3^ which highlight the importance of processing speed and accuracy for clinical integration. However, current manual analysis of OCT pullbacks is time-consuming, requires expert knowledge, and exhibits substantial inter-observer variability.^4,5^

Deep learning methods have therefore emerged as promising tools for automated OCT segmentation, enabling delineation of key structures such as lumen and plaque components.^6^ Several multi-class semantic segmentation models have reported encouraging performance for lipid and calcium plaque segmentation.^7–9^ Despite these advances, two major barriers limit clinical adoption. First, many state-of-the-art models rely on computationally intensive architectures, resulting in inference times on the order of minutes per pullback, which is incompatible with real-time decision-making during PCI. Second, highly accurate segmentation requires large volumes of pixel-wise annotated OCT data, which are costly and time-consuming to obtain. We previously introduced OCT-AID, a deep learning-based framework for multi-class OCT segmentation. However, its implementation required approximately 200 s to process a 270-frame pullback, precluding real-time use.^9^

These challenges are not unique to intravascular OCT. Across medical imaging, the deployment of deep learning models in time-critical scenarios remains a central barrier to translation.^10,11^ Models achieving strong performance in research settings frequently depend on computationally intensive architectures or large ensembles, limiting feasibility in real-time clinical settings. This mismatch between offline accuracy and operational constraints has driven growing interest in model compression techniques that preserve diagnostic performance while enabling deployment on resource-constrained hardware.^12^

Knowledge distillation offers a principled approach to addressing this problem by transferring information from a high-capacity teacher model to a smaller, more efficient student model, enabling faster inference and reduced memory usage with minimal loss in performance.^13,14^ Moreover, distillation provides a natural framework for semi-supervised learning by leveraging unlabelled data through pseudo-labelling. Our work focuses on adapting OCT segmentation to the practical constraints of near real-time use, rather than introducing a new distillation technique.

**Figure 1:**
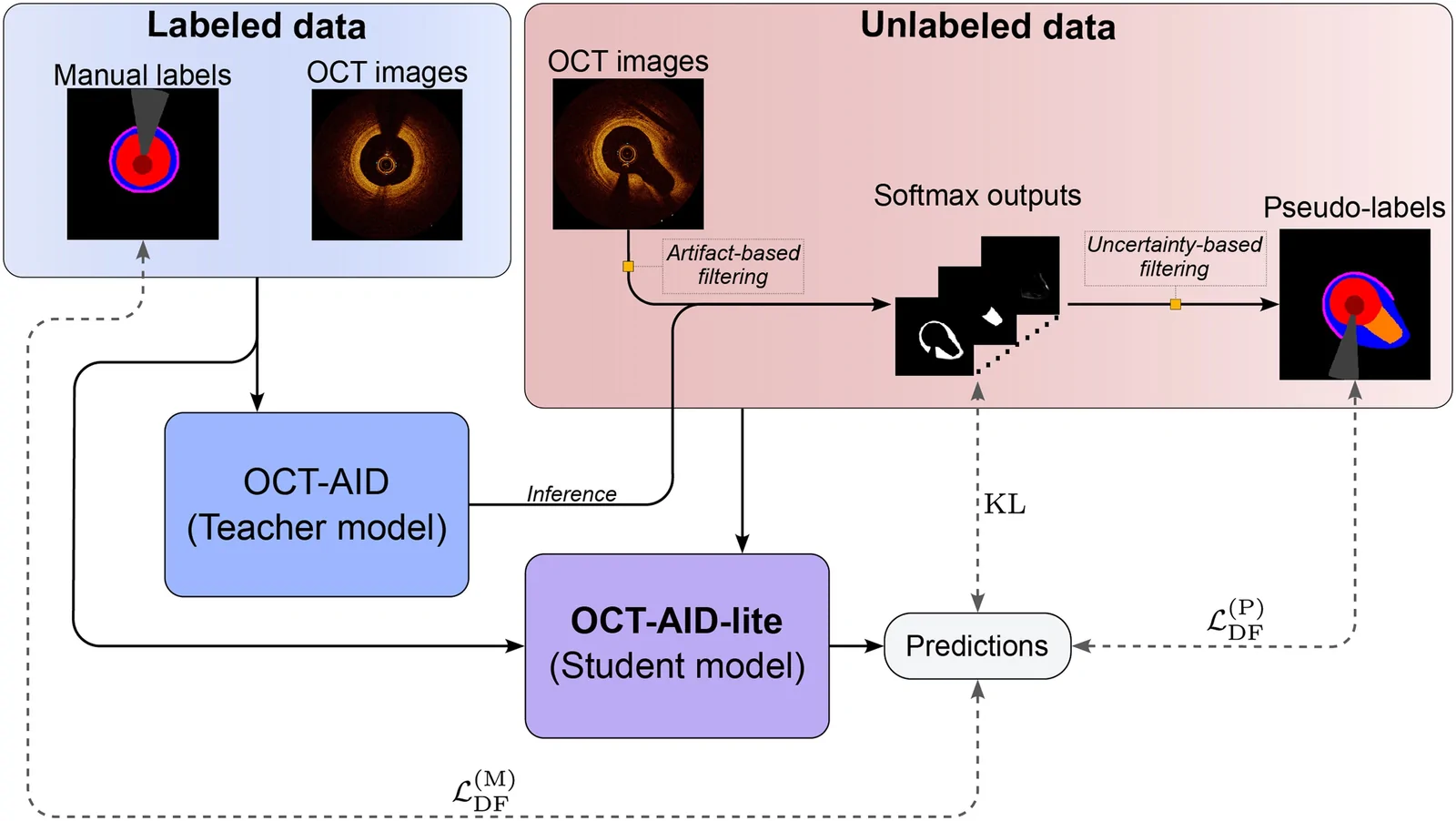
OCT-AID-lite knowledge-distillation workflow. The teacher model (OCT-AID) is first trained on labelled OCT data.^9^ During inference on unlabelled data, pseudo-labels are generated after sequential quality control steps: (1) removal of artefacted frames using an automated artefact detection algorithm^15^ and (2) exclusion of uncertain pixels based on the teacher’s softmax outputs. The filtered softmax outputs are used to obtain pseudo-labels. The lightweight student model, OCT-AID-lite, is then trained on both labelled and pseudo-labelled data. The teacher’s softmax outputs are supervised using a KL divergence loss, while hard labels (manual and pseudo-labels) are optimized using a combined Dice–Focal loss, $L_{\mathrm{DF}}^{\left(M\right)}$ and $L_{\mathrm{DF}}^{\left(P\right)}$. DF, Dice–Focal; KL, Kullback–Leibler; $L_{h}$, hard loss; $L_{s}$, soft loss; M, manual labelled data; P, pseudo-labelled data.

In this study, we extend the OCT-AID framework,^9^ by integrating response-based knowledge distillation with a semi-supervised learning strategy to enable near-real-time OCT segmentation. A high-capacity teacher model is trained on manually annotated OCT data and used to generate pseudo-labels for a large unlabelled dataset. These pseudo-labels are subsequently filtered using automated quality control based on artefact detection and uncertainty quantification. A lightweight student model, termed OCT-AID-lite, is then trained on the combined manually labelled and quality-controlled pseudo-labelled data (*Figure 1*). This approach aims to substantially reduce inference time while preserving clinically meaningful segmentation accuracy, thereby facilitating near-real-time intravascular OCT analysis in the catheterization laboratory.

## Methods

### Study design

This study used data from two multicentre studies: The PECTUS-obs study (NCT03857971) and the ORANGE registry (Optical coherence tomography: pRognostic value and Artificial iNtelliGencE; NCT07293858). PECTUS-obs is a prospective, multicentre study investigating OCT-defined high-risk plaques in fractional flow reserve (FFR)-negative non-culprit lesions and their association with major adverse cardiovascular events in patients with recent myocardial infarction. Eligible patients had at least one non-culprit lesion with intermediate stenosis (30–90% diameter, FFR > 0.80). OCT imaging was performed using the Dragonfly Optis catheter (Abbott Vascular, USA) with fluoroscopic guidance for co-registration to arterial segments. The study complied with the principles of the 1964 Declaration of Helsinki and received approval from the institutional review board or medical ethics committee at each participating centre. The study design and primary outcomes have been previously published.^16,17^ ORANGE is a prospective multicentre OCT registry conducted in the Netherlands.^18^ Pre-PCI pullbacks, acquired prior to any interventional procedure, were randomly selected for inclusion in this study.

### Dataset characteristics

This study included a total of 338 unique patients, comprising 381 OCT pullbacks with 141 816 Cartesian frames. Of these, 3855 frames were manually labelled and 137 961 frames were pseudo-labelled. All pullbacks were acquired prior to PCI, and the dataset therefore comprised no stent-containing frames; the analysis was restricted to pre-PCI plaque characterization. The manually labelled frames were split on a patient-level using a 9:1 ratio into a training set of 3466 frames (203 patients, 229 pullbacks) and a held-out test set of 389 frames (25 patients, 25 pullbacks). Stratified sampling was performed across demographic variables (age, gender, prior myocardial infarction, diabetes, hypertension, hypercholesterolaemia, and lipid-lowering therapy), dataset origin, and all segmentation classes, using maximum allowable differences of 20% for demographic variables and 5% for dataset origin and class frequencies. All pullbacks and associated frames from a given patient were assigned to a single subset to prevent data leakage.

Across the full cohort of 338 patients, 228 contributed manually labelled frames and 260 contributed pseudo-labelled frames, with 150 patients overlapping between groups. Importantly, pseudo-labels were not generated for any patient included in the held-out test set. At the pullback level (381 pullbacks), 262 pullbacks contained manually labelled frames and 294 contained pseudo-labelled frames, with 175 pullbacks overlapping. Patient baseline characteristics and frame-level class distributions for the manually labelled and pseudo-labelled datasets are summarized in *Tables 1* and *2*.

**Table 1:** Baseline characteristics of labelled and pseudo-labelled patients

Characteristic	Labelled data (*n* = 228)	Pseudo-labelled data (*n* = 260)	*P*-value
**Age (years)**	64 ± 10	64 ± 10	*0*.*82*
**Female sex, *n* (%)**	43 (18.9%)	46 (17.7%)	*0*.*60*
**Non-smoker, *n* (%)**	73 (32.3%)	91 (35.3%)	*0*.*33*
**HT, *n* (%)**	102 (44.7%)	117 (45.0%)	*1*.*00*
**DM, *n* (%)**	34 (14.9%)	36 (13.8%)	*0*.*61*
**HC, *n* (%)**	68 (30.0%)	81 (31.2%)	*0*.*70*
**Prior MI, *n* (%)**	37 (16.2%)	37 (14.2%)	*0*.*33*
**Prior PCI, *n* (%)**	37 (16.2%)	36 (13.8%)	*0*.*24*

Values are reported as mean ± SD or *n* (%).

DM, diabetes mellitus; HC, hypercholesterolaemia; HT, hypertension; MI, myocardial infarction; PCI, percutaneous coronary intervention.

**Table 2:** Frame-level class distribution across training and test sets

Class	Train^a^ (*n* = 3466)	Train^b^ (*n* = 137 961)	Test^a^ (*n* = 389)
**Guidewire**	3466 (100.0%)	137 907 (100.0%)	389 (100.0%)
**Catheter**	3466 (100.0%)	137 961 (100.0%)	389 (100.0%)
**Lumen**	3466 (100.0%)	137 961 (100.0%)	389 (100.0%)
**Intima**	3466 (100.0%)	137 961 (100.0%)	389 (100.0%)
**Media**	3291 (95.0%)	129 916 (94.2%)	368 (94.6%)
**Sidebranch**	652 (18.8%)	30 123 (21.8%)	73 (18.8%)
**Lipid**	1859 (53.6%)	74 491 (54.0%)	213 (54.8%)
**Calcium**	1162 (33.5%)	37 328 (27.1%)	133 (34.2%)
**Layered** plaque	722 (20.8%)	26 006 (18.9%)	95 (24.4%)
**Red** thrombus	157 (4.5%)	2432 (1.8%)	25 (6.4%)
**White** thrombus	250 (7.2%)	2805 (2.0%)	16 (4.1%)
**Plaque** rupture	246 (7.1%)	5001 (3.6%)	10 (2.6%)
**Microvessels**	460 (13.3%)	17 080 (12.4%)	38 (9.8%)

^a^Manually labelled frames

^b^Pseudo-labelled frames.

Frame counts (*n*) and percentages (%) indicate the presence of each class in the respective dataset, with percentages relative to the total number of frames in that set.

### Data annotation and preparation

OCT pullbacks were exported as 704 × 704 RGB Cartesian volumes (pixel spacing 9.9 µm). A circular mask (radius 352 pixels) was applied to remove watermarks, followed by greyscale conversion and z-score intensity normalization. To incorporate spatial context, each input consisted of a central frame stacked with three upstream and three downstream frames, resulting in a 7 × 704 × 704 tensor; zero padding was applied when necessary.

The reference standard annotations were created by an annotation team of trained personnel with 2–5 years of OCT experience, following contemporary guidelines.^3^ Weekly consensus meetings were held to resolve ambiguous cases. Pixel-wise annotations included 13 classes: guidewire, catheter, lumen, side branch, intima, media, lipid, calcium, layered plaque, red thrombus, white thrombus, plaque rupture, and microvessels.

### OCT-AID model

The teacher model OCT-AID is based on the nnU-Net 2D ensemble framework and has been described previously.^9,19,20^ Five-fold cross-validation was used, with each fold trained 1000 epochs using stochastic gradient descent (batch size 5) and a polynomial learning rate schedule (initial learning rate 0.01). The model employed Dice and Cross-Entropy loss with deep supervision, Leaky ReLU activations and encoder-decoder channel sizes of (32, 64, 128, 256, 512, 512, 512, 512), totalling to 46.35 million parameters per fold. Extensive on-the-fly data augmentations were applied, including geometric (rotation, scaling, mirroring), photometric (brightness and contrast adjustments), and noise-based transformations (Gaussian noise, blur, and low-resolution simulation). The final teacher consisted of an ensemble of the five trained folds.

### Knowledge distillation framework

The OCT-AID teacher model was applied to unlabelled OCT frames to generate pixel-wise probability distributions across all classes (softmax outputs). Only frames without manual annotations were treated as unlabelled inputs. Pseudo-labels were obtained by assigning each pixel to the class with the highest predicted probability (argmax); predictions were generated in a single inference pass without iterative refinement.

The student model, OCT-AID-lite, was trained using a combined distillation loss comprising supervision from both hard labels (manual and pseudo-labels) and soft labels derived from the teacher’s softmax outputs. OCT-AID-lite was trained to minimize the following objective:


(1) $$\begin{array}{c} \;L=\underset{L_{h}}{\underbrace{L_{\mathrm{DF}}^{\left(M\right)}+L_{\mathrm{DF}}^{\left(P\right)}}}+L_{s} \end{array}$$

where the hard loss $L_{h}$ is the sum of the Dice–Focal losses^21^ applied to manually labelled (*M*) and pseudo-labelled (*P*) data. The soft loss $L_{s}$ is the Kullback–Leibler (KL) divergence between teacher and student softmax outputs on pseudo-labelled data:


(2) $$L_{s}=\mathrm{KL}(p_{\mathrm{tea}}^{\left(P\right)}\;∥\;p_{\mathrm{stu}})$$

where $p_{\mathrm{tea}}^{\left(P\right)}$ and $p_{\mathrm{stu}}^{\left(P\right)}$ denote the teacher and student softmax probability distributions for the pseudo-labelled data, respectively.

#### OCT-AID-lite development and training details

OCT-AID-lite is a lightweight 2D U-Net^22^ with channel sizes (16, 32, 64, 128, 256, 256, 256, 256), two residual units per block and a symmetric decoder with transposed convolutions. Leaky ReLU activations and instance normalization were used throughout. OCT-AID-lite was trained and validated using the data splits from a single fold of the OCT-AID cross-validation. Training was performed for 1000 epochs with a batch size of 16, with the training set balanced between manually labelled and pseudo-labelled frames. Optimization used stochastic gradient descent with a polynomial learning rate schedule (initial learning rate 0.01). Data augmentations mirrored those applied during teacher training, including rotations, scaling, brightness/contrast adjustments, Gaussian noise, blur, low-resolution simulation, and axis mirroring. The final model was selected based on the highest mean validation Dice score.

#### Quality control of pseudo-labelled data

Two strategies were implemented to automatically assess the quality of OCT-AID predictions on unlabelled data prior to OCT-AID-lite training. Frame-level quality control excluded frames flagged by a previously developed artefact detection model,^15^ with detailed performance reported in the original article.

Pixel-level quality control filtered high-uncertainty pixels based on normalized softmax entropy computed from the ensemble teacher model. For each pixel, the entropy was calculated as:


(3) $$H=-\frac{1}{\mathrm{lo}g_{2}C}\overset{C}{\underset{c=1}{\sum }}\;p_{\mathrm{tea},c}^{\left(P\right)}\mathrm{lo}g_{2}p_{\mathrm{tea},c}^{\left(P\right)}$$

where *C* is the number of classes, and $p_{\mathrm{tea},c}^{\left(P\right)}$ denotes the teacher’s softmax probability for class *c* on pseudo-labelled data. Within each pseudo-labelled frame, the top 5% highest entropy pixels were removed,^23^ a threshold selected from validation set analysis comparing 0%, 5%, and 10% exclusion (see Supplementary material online, *Table S1*). Thresholds outside this range were not evaluated. The overall knowledge distillation framework is illustrated in *Figure 1*, and additional ablation experiments are provided in the Supplementary Materials (Ablation study).

### Post-processing

Post-processing was applied independently to each predicted segmentation map using connected component analysis with 4-connectivity. Connected regions smaller than predefined class-specific area thresholds were reassigned based on their 8-neighbourhood: regions were classified as background if >75% of neighbouring pixels were background, and otherwise assigned to the most frequent neighbouring non-background class. Area thresholds were 0.03 mm^2^ for background and lumen; 0.10 mm^2^ for guidewire, intima, and lipid plaque; 0.01 mm^2^ for calcium plaque, media, and side branch, consistent with prior work.^9^

### Model evaluation

#### Internal validation

Segmentation performance was evaluated using class-specific Dice scores and the 95th-percentile Hausdorff distance (HD95) as commonly reported.^24^ Dice scores were computed on (i) true-positive frames only and (ii) all frames in which the target class was present in either the reference annotation or the model prediction (true-positive, false-positive, or false-negative frames). Frame-level true positives, false positives, and false negatives were defined by the presence or absence of at least one pixel belonging to the target class; false-positive and false-negative frames were assigned a Dice score of zero.

Frame-level classification performance for lipid plaques, calcium plaques, and thin-cap fibroatheroma (TCFA) was assessed using sensitivity and specificity with 95% confidence intervals (CIs). All internal validation analyses were performed on the held-out test set. For both the teacher and student models, all internal evaluation metrics (Dice, HD95, and frame-level classification performance) were computed on the same held-out test set of manually annotated frames.

Continuous plaque metrics included lipid arc (°; angular extent around the catheter centre) and minimum fibrous cap thickness (FCT) (µm; the shortest distance from lumen to adluminal lipid border) for lipid plaques. For calcium plaques, continuous plaque metrics included maximum calcium arc (°), minimum calcium depth (µm; the shortest distance between the lumen and adluminal calcium border), and maximum calcium thickness (µm; the longest distance between the adluminal and abluminal calcium border). TCFA was defined as a lipid arc ≥90° and a minimum FCT <65 µm. Quantitative measurements are reported as median [interquartile range (IQR)], and agreement between automated and reference measurements was evaluated using Bland–Altman analysis on true-positive frames.

Comparative analyses of alternative student model architectures are reported in the Supplementary Materials (Student model architecture comparison).

#### Inference time evaluation

Inference time was evaluated for both the forward pass alone and the complete processing pipeline, which included pre- and post-processing steps. Pre-processing comprised file loading, circular masking, greyscale conversion, z-score normalization, and construction of pseudo-3D input tensors. Timing experiments were performed on eight randomly selected pullbacks, each processed five times to obtain stable estimates.

All models were evaluated under identical conditions, and runtime comparisons were restricted to models evaluated on the same hardware. Measurements were conducted on an NVIDIA A100 GPU (40 GB) with 8 CPU cores and 80 GB of RAM. To assess performance on consumer-grade hardware, additional inference experiments were conducted on a laptop (Intel Core i9, 14 CPU cores, 32 GB RAM, NVIDIA GeForce RTX 3050 GPU with 4 GB VRAM) using four CPU processes.

#### External validation

Model generalizability was assessed using an independent external dataset from a Core Lab registry (Real Solution Sp. Z o.o., Poland), comprising 29 patients (29 pullbacks, 392 frames). All patients underwent clinically indicated OCT, without restriction to non-flow-limiting lesions.

Model predictions were compared with independent manual assessments by two experienced interventional cardiologists blinded to the model outputs. Only frames with inter-observer agreement on class presence or absence were included in the analysis. For continuous plaque metrics (lipid arc, FCT, calcium arc, calcium depth, and calcium thickness), the reference standard was defined as the mean of the two observers’ measurements, and inter-observer variability was quantified by comparing their independent measurements.

### Statistical analysis

Categorical variables are presented as counts (percentages), and continuous variables as mean ± standard deviation (SD) or median (IQR). At the patient level, comparisons between labelled and pseudo-labelled datasets were performed using generalized estimating equations (GEEs) to account for repeated measures within the same patient. An exchangeable correlation structure was used; categorical variables were evaluated with a logit link, continuous outcomes with an identity link, and variables with a gamma distribution were modelled using a log link.

Inference times were compared using a paired Student’s *t*-test. Segmentation metrics were compared using paired tests selected according to distributional assumptions: the paired Student’s *t*-test for approximately normally distributed variables and the Wilcoxon signed-rank test otherwise. Frame-level sensitivity and specificity for OCT-AID-lite and OCT-AID were compared using McNemar’s test. Two-sided *P*-values <0.05 were considered statistically significant. Analyses were performed using SPSS Statistics version 27 (IBM Corp., USA).

## Results

### Internal validation

#### Segmentation performance

Pixel-wise segmentation performance of OCT-AID-lite and the teacher model OCT-AID on the held-out test set is reported in *Table 3*. Overall, OCT-AID-lite closely matched the teacher model, with marginal reductions in Dice scores and small increases in HD95 values. On true-positive frames, OCT-AID-lite demonstrated high performance for guidewire, catheter, lumen, intima, and media (Dice: 0.94–0.99), and moderate to high performance for sidebranch, lipid, and calcium (Dice: 0.76–0.78). For lipid and calcium plaques, mean Dice score differences relative to OCT-AID were 0.01 and 0.02, respectively, while the corresponding HD95 differences were 0.04 and 0.01 mm; none of these differences were statistically significant.

**Table 3:** Segmentation performance of OCT-AID-lite and OCT-AID

	OCT-AID-lite		OCT-AID		*P*-value	
Forward-pass time(s)	0.10 ± 0.03		24.22 ± 0.10		*<0.01*	
	Dice	HD95 (mm)	Dice	HD95 (mm)	Dice	HD95
**Guidewire**	0.94 ± 0.04	0.02 (0.00–0.06)	0.94 ± 0.06	0.02 (0.00–0.06)	*0*.*59*	*0*.*12*
**Catheter**	0.99 ± 0.01	0.00 (0.00–0.00)	0.99 ± 0.01	0.00 (0.00–0.00)	***<0***.***01***	*1*.*00*
**Lumen**	0.98 ± 0.03	0.00 (0.00–0.00)	0.98 ± 0.04	0.00 (0.00–0.00)	*0*.*78*	*0*.*80*
**Sidebranch**	0.78 ± 0.25	0.07 (0.02–0.23)	0.79 ± 0.27	0.07 (0.01–0.21)	*0*.*61*	*0*.*96*
**Intima**	0.86 ± 0.11	0.07 (0.02–0.18)	0.87 ± 0.11	0.06 (0.01–0.18)	***<0***.***01***	***0***.***01***
**Media**	0.79 ± 0.16	0.08 (0.02–0.47)	0.81 ± 0.17	0.07 (0.01–0.43)	***<0***.***01***	*0*.*11*
**Lipid**	0.76 ± 0.16	0.19 (0.09–0.50)	0.77 ± 0.18	0.15 (0.07–0.39)	*0*.*46*	*0*.*25*
**Calcium**	0.77 ± 0.22	0.11 (0.03–0.37)	0.79 ± 0.21	0.10 (0.02–0.35)	*0*.*22*	*0*.*65*
**Layered** plaque	0.50 ± 0.29	0.48 (0.19–0.87)	0.58 ± 0.31	0.59 (0.18–0.99)	*0*.*75*	*0*.*33*
**Red** thrombus	0.65 ± 0.24	0.19 (0.09–0.38)	0.55 ± 0.31	0.25 (0.03–0.60)	*0*.*34*	*0*.*49*
**White** thrombus	0.39 ± 0.31	1.00 (0.10–1.57)	0.39 ± 0.35	0.88 (0.42–1.01)	*0*.*39*	*1*.*00*
**Plaque** rupture	0.67 ± 0.30	0.17 (0.02–0.40)	0.64 ± 0.38	0.33 (0.01–0.57)	*0*.*71*	*0*.*48*
**Microvessels**	0.66 ± 0.21	0.09 (0.04–0.61)	0.74 ± 0.18	0.04 (0.01–0.91)	***0***.***018***	*0*.*54*

Performance reported as mean ± SD for inference time and Dice scores on true-positive frames, and median (IQR) for HD95. Bold *P*-values indicate statistical significance (*P*  *<*  *0.05*).

HD, Hausdorff distance; IQR, interquartile range; SD, standard deviation.

Performance was lower and more variable for morphologically complex and less prevalent structures, including layered plaque, red thrombus, white thrombus, plaque rupture, and microvessels (Dice: 0.39–0.67). For these classes, OCT-AID-lite exhibited performance comparable to OCT-AID. Dice scores computed on all frames are reported in Supplementary material online, *Table S2*. Restricting training to non-overlapping subsets of training patients (manual vs. pseudo-labelled) yielded comparable performance (see Supplementary material online, *Table S3*). As OCT-AID is an ensemble model, single-fold OCT-AID inference time and segmentation performance are presented in Supplementary material online, *Table S4*.

**Figure 2:**
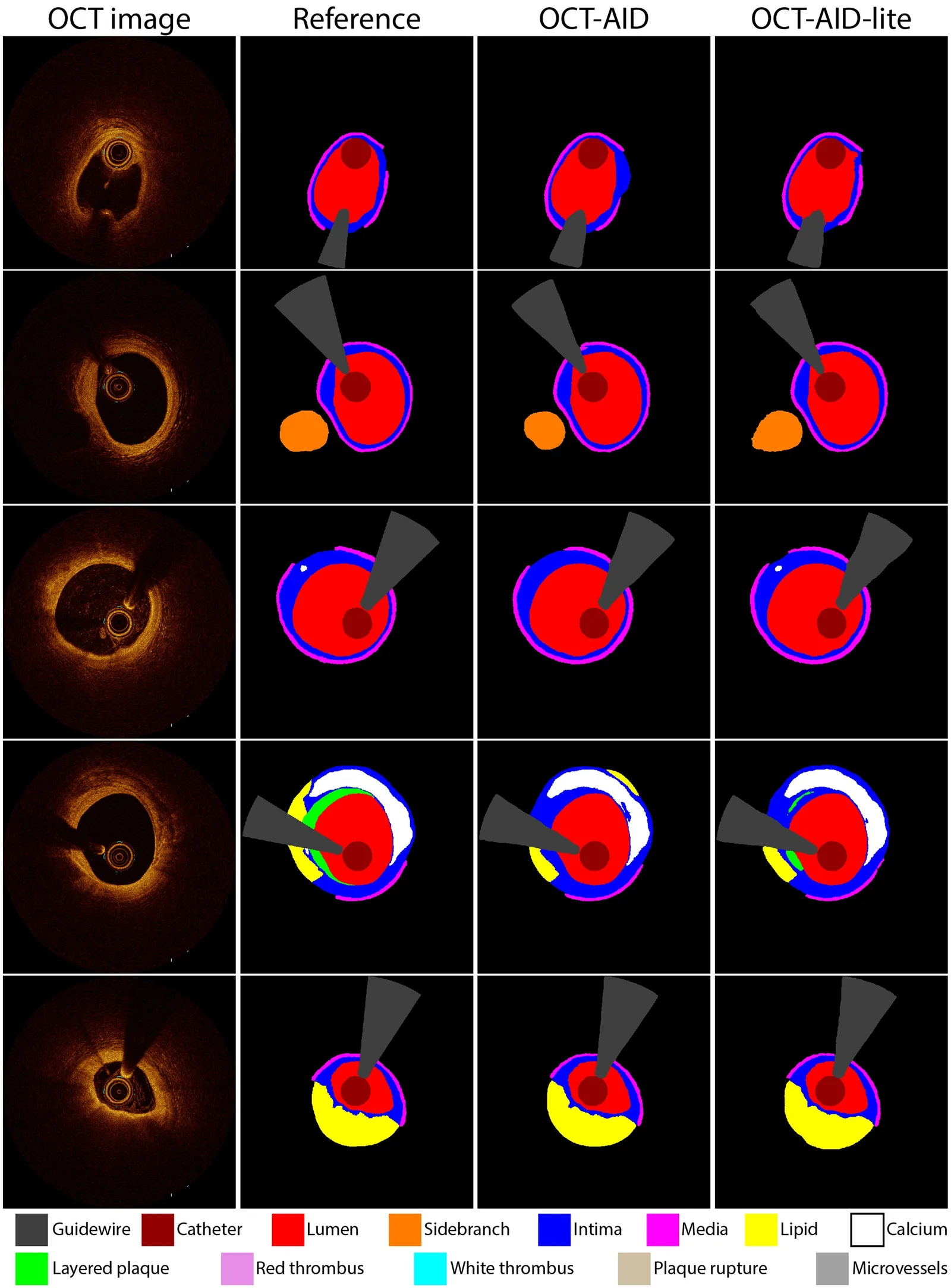
Qualitative comparison of segmentation predictions from OCT-AID and OCT-AID-lite. Each row shows the OCT image, the reference annotation, and the corresponding model predictions from OCT-AID and OCT-AID-lite, with class labels indicated by the colour-coded legend.

Qualitative evaluation demonstrated accurate delineation of lumen, intima, and media contours by OCT-AID-lite, closely matching the reference standard (*Figure 2*). Guidewire and catheter were segmented with precise boundaries, whereas segmentation of more complex structures, such as layered plaque, was less consistent and occasionally incomplete. Additional challenging cases and representative segmentation errors are shown in Supplementary material online, *Figure S1*. Full pullback comparisons between OCT-AID-lite and OCT-AID are provided in Supplementary material online, *Videos S1 and S2*.

#### Classification performance

Frame-level classification performance for lipid, calcium, and TCFA is summarized in *Table 4*. OCT-AID-lite achieved high sensitivity for lipid plaque identification (98.1%, 95% CI: 95.3–99.3%), with moderate specificity (74.4%, 95% CI: 67.5–80.3%). For calcium plaque, both sensitivity (89.5%, 95% CI: 83.1–93.6%) and specificity (87.5%, 95% CI: 82.9–91.0%) were well balanced, with severity-stratified performance across <90°, 90–180°, and >180° arc bins reported in Supplementary material online, *Table S5*.

**Table 4:** Classification performance of OCT-AID-lite and OCT-AID

Class	OCT-AID-lite		OCT-AID		*P*-value
	Sensitivity (%)	Specificity (%)	Sensitivity (%)	Specificity (%)	Sens./Spec.
**Lipid**	98.1 (95.3–99.3)	74.4 (67.5–80.3)	96.7 (93.4–98.4)	80.7 (74.2–85.8)	0.38/**0.013**
**Calcium**	89.5 (83.1–93.6)	87.5 (82.9–91.0)	87.2 (80.5–91.9)	91.0 (86.9–93.9)	0.45/0.064
**TCFA**	32.1 (17.9–50.7)	100.0 (98.9–100.0)	39.3 (23.6–57.6)	100.0 (98.9–100.0)	0.50/-^a^

^a^ *P*-value not obtainable due to a constant specificity of 100% in both models, precluding McNemar’s test.

Sensitivity and specificity are reported for each class with 95% CI. Bold *P*-values indicate statistical significance (*P*  *<* 0.05).

CI, confidence interval; TCFA, thin-cap fibroatheroma.

Compared with OCT-AID, OCT-AID-lite showed slightly higher sensitivity for lipid (+1.4%, *P* = *0.38*) and calcium plaques (+2.3%, *P* = *0.45*), without statistically significant differences, and lower specificity for lipid (−6.3%, *P* = *0.013*) and calcium plaques (−3.5%, *P* = *0.064*). Sensitivity and specificity for all 13 classes are reported in Supplementary material online, *Table S6*.

Sensitivity for TCFA identification was low for both OCT-AID-lite (32.1%, 95% CI: 17.9–50.7%) and OCT-AID (39.3%, 95% CI: 23.6–57.6%; *P* = *0.50*), while specificity was 100% (95% CI: 98.9–100.0%) for both models. Error decomposition showed that OCT-AID-lite missed 19 TCFA frames and OCT-AID missed 17, with 1 miss in each model due to predicted lipid arc <90° and all remaining misses due to predicted fibrous cap thickness ≥65 µm; no cases were attributable to missed lipid plaques. Corresponding Bland–Altman analyses for all TCFA frames based on the reference standard, including false negatives, are provided in Supplementary material online, *Figure S2*.

#### Plaque quantification

**Figure 3:**
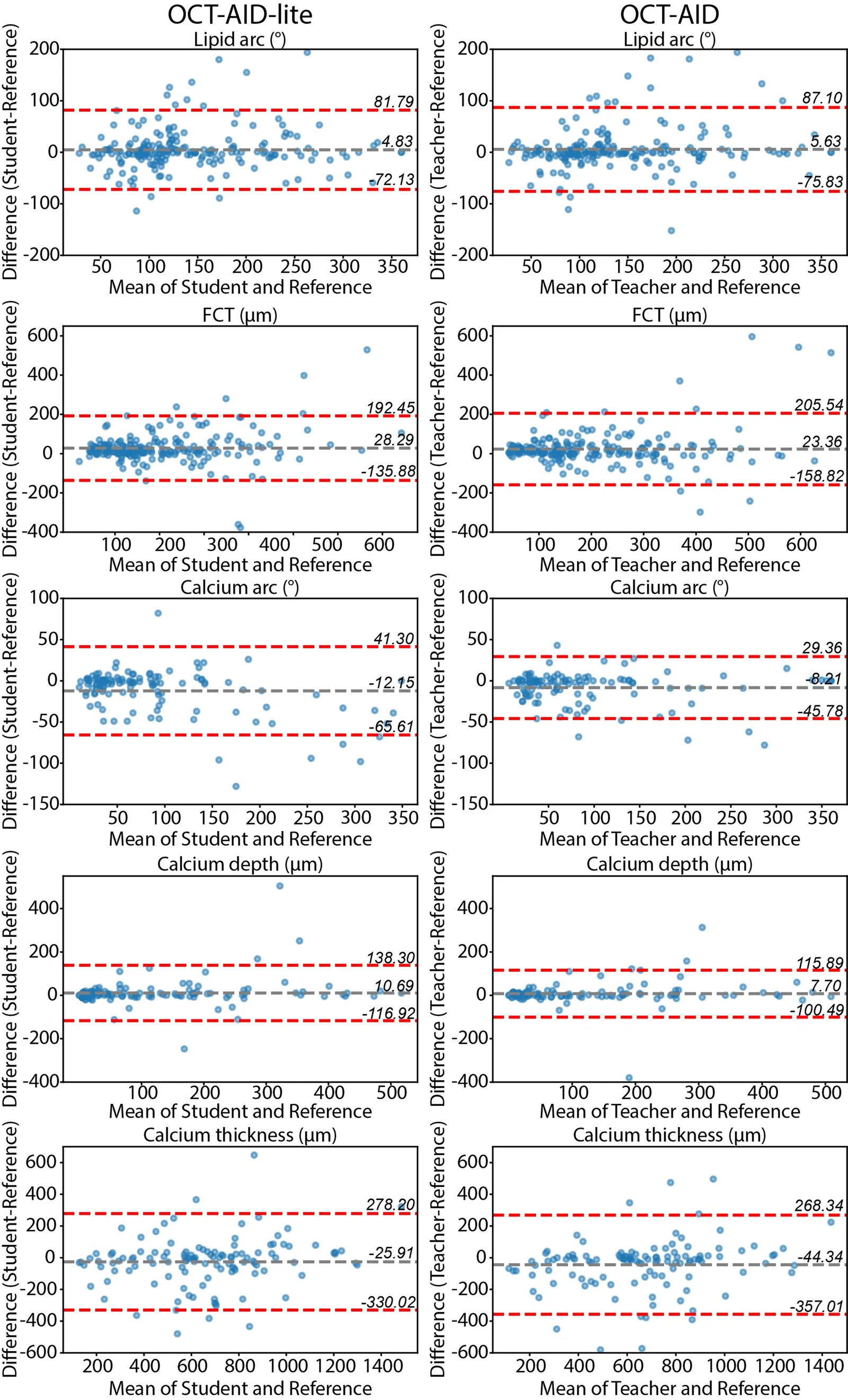
Bland–Altman analysis of OCT-AID-lite and OCT-AID for continuous lipid and calcium plaque metrics. The grey line indicates the mean difference between paired measurements, while the red lines denote the limits of agreement (mean ± 1.96 SD of the differences). SD, standard deviation.

On 209 lipid-positive frames, OCT-AID-lite measured a median (IQR) lipid arc of 125° (90–188°) compared with 116° (91–176°) for the reference standard. The median minimum FCT was 170 µm (111–286 µm) compared with 156 µm (94–276 µm). Bland–Altman analysis (*Figure 3*, first column) showed small systematic biases for both lipid arc (4.83°) and FCT (28.29 µm). The limits of agreement were wider for FCT than for lipid arc, indicating greater variability in FCT estimation. OCT-AID yielded comparable results, with similar biases and limits of agreement (*Figure 3*, second column).

On 119 calcium-positive frames, OCT-AID-lite measured a median (IQR) maximum calcium arc of 63° (31–111°) compared with 66° (37–123°) for the reference standard. The median minimum calcium depth was 81 µm (20–197 µm) compared with 70 µm (14–180 µm), and the median maximum calcium thickness was 607 µm (444–825 µm) vs. 682 µm (456–847 µm), respectively. Bland–Altman analysis showed small biases across all calcium metrics (calcium arc: −12.15°; calcium depth: 10.69 µm; calcium thickness: −25.91 µm). The widest limits of agreement were observed for calcium thickness. OCT-AID demonstrated comparable bias magnitudes and agreement limits. Bland-Altman analyses, including true-positive, false-positive, and false-negative frames, are provided in Supplementary material online, *Figure S3*.

### Inference time evaluation

On an NVIDIA A100 GPU, OCT-AID-lite achieved a mean forward-pass time of 0.10 ± 0.03 s per 540-frame pullback, compared with 24.22 ± 0.10 s for OCT-AID under identical conditions (*P*  *<*  *0.01*). Including pre-processing (1.74 ± 0.94 s) and post-processing (3.67 ± 0.28 s), total pipeline time for OCT-AID-lite was 5.50 ± 1.06 s per pullback, compared with 30.62 ± 1.01 s for OCT-AID (*P*  *<*  *0.01*).

On consumer hardware (laptop with NVIDIA GeForce RTX 3050), OCT-AID-lite achieved a forward-pass time of 5.13 ± 2.68 s and a total processing time of 22.82 ± 7.17 s per pullback, with a 95th-percentile latency of 35.81 s. Under these conditions, OCT-AID required 143.48 ± 1.46 s for the forward-pass (*P*  *<*  *0.001*), with a total processing time of 170.61 ± 11.68 s. All settings were identical except batch size (64 for OCT-AID-lite; 16 for OCT-AID due to memory constraints).

### External validation

External validation results based on consensus are reported in *Table 5*, with the corresponding individual observer and model-vs.-observer agreement tables provided in Supplementary material online, *Tables S7–S10*.

**Table 5:** External validation performance of OCT-AID-lite

Class	Inter-observer Kappa/ICC^a^	Frames with consensus^b^	Prevalence	TP frames	Accuracy (%)	Sensitivity (%)	Specificity (%)	PPV (%)	NPV (%)	External testing Kappa/ICC^a^
**Lipid plaque**	0.535 (0.451–0.619)	306	104 (34.0%)	96 (92.3%)	85.9 (81.1–89.6)	92.3 (85.6–96.1)	82.7 (76.9–87.3)	73.3 (65.1–80.1)	95.4 (91.2–97.7)	0.705 (0.654–0.756)
**Arc**	0.516 (0.359–0.644)	—	—	—	—	—	—	—	—	0.866 (0.763–0.920)
**Minimum FCT**	0.392 (0.126–0.586)	—	—	—	—	—	—	—	—	0.782 (0.673–0.855)
**Calcium plaque**	0.905 (0.860–0.950)	376	115 (30.6%)	107 (93.0%)	92.3 (89.1–94.6)	93.0 (86.9–96.4)	92.0 (88.0–94.7)	83.6 (76.2–89.0)	96.8 (93.8–98.4)	0.824 (0.785–0.862)
**Arc**	0.772 (0.566–0.869)	—	—	—	—	—	—	—	—	0.906 (0.715–0.956)
**Depth**	0.708 (0.569–0.802)	—	—	—	—	—	—	—	—	0.880 (0.824–0.918)
**Thickness**	0.803 (0.715–0.864)	—	—	—	—	—	—	—	—	0.668 (0.509–0.775)

^a^Cohen’s kappa was used for categorical variables (lipid plaque, calcium plaque), whereas the ICC was used for continuous variables (lipid arc, minimum FCT, calcium arc, calcium depth, and calcium thickness).

^b^Frames for which the independent observers agreed on the presence or absence of the evaluated feature.

Performance of OCT-AID-lite on external validation frames for lipid and calcium plaques, compared with consensus reference. Metrics are reported as value (95% CI).

CI, confidence interval; FCT, fibrous cap thickness; ICC, intraclass correlation coefficient; NPV, negative predictive value; PPV, positive predictive value; TP, true positive.

For lipid plaque presence, OCT-AID-lite achieved a κ of 0.705 (95% CI: 0.654–0.756), exceeding the average inter-observer agreement between the two expert cardiologists (κ = 0.535, 95% CI: 0.451–0.619). Frame-level accuracy was 85.9%, with a sensitivity of 92.3% and a specificity of 82.7%. Continuous measurements showed strong agreement for lipid arc (ICC = 0.764) and good agreement for minimum FCT (ICC = 0.642). Agreement for lipid arc was observer-dependent, with agreement below the inter-observer level for Observer 1 and above the inter-observer level for Observer 2 (see Supplementary material online, *Tables S7–S10*).

For calcium plaque presence, OCT-AID-lite achieved a κ of 0.824 (95% CI: 0.785–0.862), slightly below inter-observer agreement (κ = 0.905, 95% CI: 0.860–0.950). Frame-level accuracy was 92.3%, with a sensitivity of 93.0% and a specificity of 92.0%. Agreement was strong for calcium arc (ICC = 0.888) and calcium depth (ICC = 0.776), and moderate for calcium thickness (ICC = 0.498). We additionally evaluated the teacher model on the external dataset using the same consensus-based reference standard (see Supplementary material online, *Table S11*), providing a direct comparison of teacher and student performance under identical conditions.

## Discussion

This study demonstrates the feasibility of combining knowledge distillation with semi-supervised learning to enable near real-time OCT segmentation. The proposed OCT-AID-lite model achieved two key outcomes: (1) a significant reduction in inference time, enabling near real-time clinical use and (2) segmentation and plaque quantification performance closely aligned with the reference standard and the high-capacity teacher model. Importantly, this work focuses on re-engineering OCT segmentation for near-real-time clinical deployment, integrating artefact and uncertainty outputs into a lightweight pipeline rather than introducing a new distillation algorithm.

OCT-AID-lite reduced the forward-pass inference time for a 540-frame pullback from 24.22 to 0.10 s, while reducing total pipeline time from 30.62 to 5.50 s. This acceleration is critical for clinical adoption as prolonged diagnostic analysis remains a major barrier to routine intravascular OCT use.^4^ Prior studies reported considerably longer inference times, including 21.4 s for a 270-frame pullback^7^ and 180–300 s for 271–540 frames.^8^ While direct comparisons are limited by differences in hardware, these findings illustrate the potential of knowledge distillation to achieve near real-time performance without substantial loss of segmentation accuracy for most classes. We additionally evaluated performance on consumer-grade hardware to provide a conservative benchmark, although ongoing advances in dedicated AI inference hardware suggest that substantially more powerful systems are likely to become standard in clinical environments.

Segmentation performance for lipid and calcium plaques was comparable to previously reported results, with Dice scores of 0.76 and 0.77, respectively.^7,8^ Direct cross-study comparison remains challenging due to differences in datasets and reporting conventions, particularly with respect to whether metrics are computed on true-positive frames only or on all frames. For transparency, we report results using both approaches. The modest performance trade-off relative to the teacher model is consistent with the expected effects of model compression,^25^ and qualitative evaluation confirmed close agreement between OCT-AID-lite, OCT-AID, and the reference standard for most plaque structures (*Figure 2*; Supplementary material online, *Videos S1 and S2*). OCT-AID-lite shows high sensitivity for both lipid and calcium, but while calcium specificity remains strong, lipid specificity is only moderate, leading to more false-positive lipid detections. Notably, pseudo-labels were derived solely from training patients, and restricting the analysis to non-overlapping training patients (i.e. no pseudo-labels from manually labelled patients) yielded similar performance, indicating gains mainly reflect increased data diversity and robustness.

Continuous plaque metrics derived from OCT-AID-lite showed small mean differences relative to OCT-AID and no systematic over- or underestimation across metrics. Bland–Altman analysis revealed wider limits of agreement for FCT and calcium thickness, reflecting the intrinsic difficulty of accurately delineating thin fibrous caps and abluminal calcium borders in the presence of OCT signal attenuation. Given the moderate inter-observer agreement reported for these measurements, the level of performance that can reasonably be expected from the model is limited by inherent human variability. As such, discrepancies between model predictions and reference measurements should be interpreted in the context of this variability, particularly for frame-level measurements. Consequently, while OCT-AID-lite enables reliable plaque characterization at the lesion and patient level, individual frame-level measurements should be interpreted with caution. Sensitivity for TCFA detection was low for both OCT-AID-lite and OCT-AID, indicating a task-level limitation rather than an effect of distillation. TCFA classification relies on compound threshold criteria, such that small measurement errors in lipid arc or fibrous cap thickness can disproportionately affect classification. Accordingly, binary TCFA identification is not yet clinically actionable, and the model’s practical value in real-world PCI scenarios lies primarily in its continuous measurements rather than categorical TCFA labels. Continuous measurements, which demonstrated consistent agreement with the reference standard, may therefore offer greater clinical utility than binary TCFA classification.

On the external validation dataset, OCT-AID-lite achieved agreement with individual expert observers that was comparable to average inter-observer agreement for lipid and calcium plaque identification. The observer-dependent differences observed for lipid arc measurements likely reflect the inherent variability of manual plaque characterization. Future studies incorporating a larger number of expert observers are warranted to provide a more robust benchmark against which to evaluate automated model outputs. Continuous plaque metrics showed generally good agreement for arc and depth, while agreement for calcium thickness showed greater variability, consistent with reduced OCT signal penetration in deeper vessel wall structures.

The computational efficiency of OCT-AID-lite supports the technical feasibility of integration into clinical OCT workflows. With 10.8 million parameters and inference times compatible with both console-based and consumer-grade hardware, OCT-AID-lite has the potential to be integrated as on-device software within existing OCT systems. However, its performance relative to existing vendor software remains to be established. Nevertheless, OCT-AID-lite may provide additional value through automated segmentation and quantification of plaque characteristics that are not routinely covered by current vendor software. We acknowledge that nnU-Net continues to evolve as a strong and widely adopted state-of-the-art baseline for medical image segmentation, and our teacher model is based on the most recent nnU-Net v2 framework to ensure that OCT-AID-lite is distilled from a competitive and up-to-date reference.

### Limitations

This study presents several limitations. First, segmentation performance was lower for rare and morphologically complex classes, reflecting limited training examples and high structural variability. Similar limitations have been reported previously^9^ and were also observed for the teacher model, suggesting that these challenges arise from task complexity and data scarcity rather than knowledge distillation. Frame-level performance metrics were therefore additionally reported for these classes (see Supplementary material online, *Table S5*). Despite stratified sampling, modest imbalances persisted for the rarest classes due to their very low prevalence; larger datasets will be important to mitigate this limitation. Future work may also benefit from temporal modelling approaches, such as 3D architectures that better incorporate longitudinal context, to improve spatial regularization and reduce frame-to-frame variability for these rare classes.

Second, inter-observer variability in pixel-wise manual annotations was not assessed on the test set. Future studies incorporating multiple independent annotators would help distinguish model error from intrinsic ambiguity in OCT interpretation.

Third, although comparison with the vendor’s automated analysis (e.g. Ultreon software) would provide valuable context for deployment-oriented evaluation, these console-generated outputs were not retrievable retrospectively for the cases included in this study. Because the dataset consisted exclusively of pre-PCI pullbacks, the model has not been evaluated on post-stent OCT imaging, and its generalization to stent-containing frames remains untested. Finally, validation across additional OCT systems and broader clinical settings is required to further establish generalizability.

### Conclusion

The proposed OCT-AID-lite model, developed using knowledge distillation, achieves substantially faster inference while maintaining clinically relevant segmentation and quantification performance. OCT-AID-lite therefore provides automated near real-time OCT segmentation, while improved TCFA assessment will require further model development and larger datasets.

## Supplementary Material

ztag138_Supplementary_Data

## Data Availability

The datasets used and/or analysed during the current study are available from the corresponding author on reasonable request.

## Lead author biography

Ruben van der Waerden is a PhD student at the Department of Cardiology, Radboud University Medical Center (Nijmegen, The Netherlands), and the qurAI group (Amsterdam, the Netherlands), under the supervision of Prof. Niels van Royen and Prof. Ivana Išgum, respectively. His research, embedded within the CARA Lab, focuses on developing AI-driven techniques for analysing intracoronary optical coherence tomography images. He specializes in vessel wall segmentation and plaque quantification, aiming to enhance diagnostic accuracy.
