# Supplementary material for: Near real-time multi-class segmentation for intravascular optical coherence tomography using knowledge distillation
Source: Eur Heart J Digit Health. 2026 Sep 2;7(8):ztag138. doi: 10.1093/ehjdh/ztag138 (PMC13556850; doi:10.1093/ehjdh/ztag138)
Supplement: Supplementary file 1 [file ztag138_supplementary_data.zip › 20260716_SupplementalMaterial.docx]

Supplemental material

Content

[Ablation study	4](#_Toc234921518)

[Methods	4](#_Toc234921519)

[Results	4](#_Toc234921520)

[Student model architecture comparison	6](#_Toc234921521)

[Methods	6](#_Toc234921522)

[Results	6](#_Toc234921523)

[Supplemental Figures and Videos	7](#_Toc234921524)

[Supplemental Figure 1 – Qualitative comparison of segmentation errors from OCT‑AID and OCT‑AID‑lite	7](#_Toc234921525)

[Supplemental Figure 2 – Bland–Altman analysis for TCFA frames showing true positives and false‑negative cases stratified by error mechanism.	8](#_Toc234921526)

[Supplemental Figure 3 - Bland–Altman analysis of OCT-AID-lite and OCT-AID for continuous lipid and calcium plaque metrics including TP, FP, and FN frames	9](#_Toc234921527)

[Supplemental Figure 4 - Qualitative comparison of segmentation predictions from OCT‑AID‑lite, DeepLabv3^+^ and SegFormer	10](#_Toc234921528)

[Supplemental Video 1	11](#_Toc234921529)

[Supplemental Video 2	11](#_Toc234921530)

[Supplemental Tables	12](#_Toc234921531)

[Supplemental Table 1 – Entropy exclusion sensitivity analysis	12](#_Toc234921532)

[Supplemental Table 2 – Segmentation performance OCT-AID-lite and OCT-AID for all classes	13](#_Toc234921533)

[Supplemental Table 3 – Segmentation performance OCT-AID-lite using non-overlapping patient subsets	14](#_Toc234921534)

[Supplemental Table 4 - Segmentation performance single-fold OCT-AID	15](#_Toc234921535)

[Supplemental Table 5 – Severity‑stratified calcium arc classification	16](#_Toc234921536)

[Supplemental Table 6 – Classification performance OCT-AID-lite and OCT-AID for all classes	17](#_Toc234921537)

[Supplemental Table 7 – Interobserver agreement for observer 1 vs. observer 2	18](#_Toc234921538)

[Supplemental Table 8 – Interobserver agreement for observer 2 vs. observer 1	19](#_Toc234921539)

[Supplemental Table 9 – Interobserver agreement for observer 1 vs. OCT-AID-lite	20](#_Toc234921540)

[Supplemental Table 10 – Interobserver agreement for observer 2 vs. OCT-AID-lite	21](#_Toc234921541)

[Supplemental Table 11 – External validation performance of OCT-AID	22](#_Toc234921542)

[Supplemental Table 12 - Ablation study of quality‑control components and loss functions for lipid and calcium segmentation	23](#_Toc234921543)

[Supplemental Table 13 - True‑positive Dice scores across ablation experiments and the final OCT‑AID‑lite model	24](#_Toc234921544)

[Supplemental Table 14 - Dice scores across ablation experiments and the final OCT‑AID‑lite model	25](#_Toc234921545)

[Supplemental Table 15 - Performance comparison on true-positive frames between OCT-AID-lite, DeepLabv3^+^ and SegFormer	26](#_Toc234921546)

[Supplemental Table 16 - Performance comparison between OCT-AID-lite, DeepLabv3^+^ and SegFormer	27](#_Toc234921547)

[References	28](#_Toc234921548)

## Ablation study

### Methods

We investigated the impact of using unlabeled data by varying the loss function and applying quality‑control strategies. The influence of different supervision strategies was assessed by comparing configurations of the hard and soft loss components (Equation 1). The Dice–Focal loss was applied either to manually labeled data only or to both manual and pseudo‑labeled data. The soft loss was defined using either KL divergence^1^ or mean squared error (MSE)^2^, computed between teacher and student softmax outputs. All loss components were weighted equally. The effect of quality control was evaluated through experiments with and without the frame‑ and pixel‑level filtering strategies described in the main Methods section.

### Results

Ablation results for lipid and calcium plaque segmentation are summarized in Supplemental Table 12. Dice scores for all 13 classes across all ablation experiments are reported in Supplemental Tables 13 and 14.

Three main findings were observed. First, incorporating pseudo‑labeled data consistently improved performance. Training on manually labeled data alone (Ablation 1) yielded Dice scores of 0.71 (lipid) and 0.66 (calcium). Adding pseudo labels (Ablation 2) further improved performance. This trend is consistent across all subsequent configurations, where models trained with pseudo labels (Ablations 5-6) outperform their counterparts without pseudo labels (Ablations 3-4). Second, KL divergence outperformed MSE in all settings (Ablation 4 vs. 3; Ablation 6 vs. 5), with the largest difference for calcium plaque. Third, the best configuration without quality control (Ablation 6) approached OCT‑AID‑lite performance, indicating that the combined loss and pseudo‑label strategy accounts for most of the gain.

Artifact filtering removed 12,954 frames; uncertainty filtering removed the top 5% highest‑entropy pixels. Artifact filtering alone (Ablation 7) achieved Dice 0.74 for both classes; uncertainty filtering alone (Ablation 8) achieved 0.76 and 0.77. Combining both strategies matched uncertainty‑only performance and provided additional protection against artifacted frames. Improvements over the unfiltered configuration (Ablation 9) were modest and not statistically significant (p = 0.90 and 0.98).

## Student model architecture comparison

### Methods

The proposed OCT-AID-lite model was compared with alternative student model architectures, including DeepLabv3^+3^ and SegFormer with a MiT-B1 encoder^4^, selected for their inference efficiency and segmentation accuracy. All models were trained using identical hard and soft loss configurations and the same pseudo-label quality control procedures, including frame-level artifact exclusion and pixel-level uncertainty filtering. Segmentation performance and inference time were evaluated for each model to assess both accuracy and computational efficiency.

### Results

Performance comparison with alternative student architectures is shown in Supplemental Table 15. DeepLabv3+ and SegFormer achieved Dice scores comparable to OCT‑AID‑lite for several classes; however, statistically significant differences were observed for intima and media for both architectures, and for lipid plaque in DeepLabv3+ (all p < 0.01). Calcium plaque segmentation did not differ significantly between models. For rare and morphologically complex classes, DeepLabv3+ failed to predict any instances of layered plaque, red thrombus, white thrombus, plaque rupture, or microvessels, while SegFormer predicted only layered plaque and plaque rupture. In contrast, OCT‑AID‑lite consistently predicted all 13 classes. Forward‑pass inference times were similar across architectures (0.10–0.13 s); although small differences were statistically significant (p < 0.01), they are unlikely to be clinically meaningful. Qualitative comparisons are shown in Supplemental Figure 4, and Dice scores across all frames are reported in Supplemental Table 16.

## Supplemental Figures and Videos

### Supplemental Figure 1 – Qualitative comparison of segmentation errors from OCT‑AID and OCT‑AID‑lite

Each row shows the OCT image, the reference annotation, and the corresponding model predictions from OCT-AID and OCT-AID-lite, with class labels indicated by the color-coded legend.

### Supplemental Figure 2 – Bland–Altman analysis for TCFA frames showing true positives and false‑negative cases stratified by error mechanism.

The gray line indicates the mean difference between paired measurements, and the red lines denote the limits of agreement (mean ± 1.96 SD of the differences). Analyses include true‑positive TCFA frames and false‑negative cases, color‑coded by error mechanism (lipid arc < 90° or fibrous cap thickness ≥ 65 µm).

*SD, standard deviation.*

### Supplemental Figure 3 - Bland–Altman analysis of OCT-AID-lite and OCT-AID for continuous lipid and calcium plaque metrics including TP, FP, and FN frames

The gray line indicates the mean difference between paired measurements, and the red lines denote the limits of agreement (mean ± 1.96 SD of the differences). Analyses include true positive (blue), false negative (red), and false positive (yellow) frames.

*SD, standard deviation.*

### Supplemental Figure 4 - Qualitative comparison of segmentation predictions from OCT‑AID‑lite, DeepLabv3^+^ and SegFormer

Each row shows the OCT image, the reference annotation, and the corresponding model predictions from OCT‑AID‑lite, DeepLabv3^+^, and SegFormer, with class labels indicated by the color‑coded legend.

### Supplemental Video 1

### Supplemental Video 2

## Supplemental Tables

### Supplemental Table 1 – Entropy exclusion sensitivity analysis

| Class | No entropy exclusion | 5% entropy exclusion | 10% entropy exclusion |
| --- | --- | --- | --- |
| Guidewire | 0.94±0.11 | 0.94±0.11 | 0.93±0.12 |
| Catheter | 0.99±0.01 | 0.99±0.01 | 0.99±0.01 |
| Lumen | 0.98±0.05 | 0.98±0.05 | 0.98±0.06 |
| Sidebranch | 0.55±0.43 | 0.55±0.43 | 0.53±0.43 |
| Intima | 0.88±0.11 | 0.88±0.11 | 0.87±0.11 |
| Media | 0.79±0.21 | 0.79±0.21 | 0.77±0.22 |
| Lipid | 0.63±0.33 | 0.63±0.33 | 0.62±0.34 |
| Calcium | 0.61±0.38 | 0.64±0.37 | 0.60±0.38 |
| Layered Plaque | 0.29±0.38 | 0.31±0.39 | 0.26±0.36 |
| Red Thrombus | -^a^ | 0.20±0.32 | 0.20±0.33 |
| White Thrombus | -^a^ | 0.18±0.33 | 0.19±0.31 |
| Plaque Rupture | 0.34±0.41 | 0.38±0.42 | 0.38±0.42 |
| Microvessels | 0.35±0.36 | 0.22±0.32 | 0.20±0.31 |

^a^ Dice score not defined because the model did not predict any instances of this class.

Validation‑set Dice scores comparing 0%, 5%, and 10% entropy‑based pixel exclusion.

### Supplemental Table 2 – Segmentation performance OCT-AID-lite and OCT-AID for all classes

|  | OCT-AID-lite | | OCT-AID | | p-value | |
| --- | --- | --- | --- | --- | --- | --- |
| Forward-pass  time (s) | 0.10±0.03 | | 24.22±0.10 | | **<0.01** | |
|  | **Dice** | **HD95 (mm)** | **Dice** | **HD95 (mm)** | **Dice** | **HD95** |
| Guidewire | 0.94±0.04 | 0.02 (0.00-0.06) | 0.94±0.06 | 0.02 (0.00-0.06) | *0.59* | *0.12* |
| Catheter | 0.99±0.01 | 0.00 (0.00-0.00) | 0.99±0.01 | 0.00 (0.00-0.00) | ***<0.01*** | *1.00* |
| Lumen | 0.98±0.03 | 0.00 (0.00-0.00) | 0.98±0.04 | 0.00 (0.00-0.00) | *0.78* | *0.80* |
| Sidebranch | 0.48±0.43 | 0.07 (0.02-0.23) | 0.57±0.42 | 0.07 (0.01-0.21) | *0.59* | *0.96* |
| Intima | 0.86±0.12 | 0.07 (0.02-0.18) | 0.87±0.11 | 0.06 (0.01-0.18) | ***<0.01*** | ***0.01*** |
| Media | 0.77±0.20 | 0.08 (0.02-0.47) | 0.79±0.21 | 0.07 (0.01-0.43) | ***<0.01*** | *0.11* |
| Lipid | 0.62±0.33 | 0.19 (0.09-0.50) | 0.64±0.33 | 0.15 (0.07-0.39) | *0.64* | *0.25* |
| Calcium | 0.55±0.39 | 0.11 (0.03-0.37) | 0.59±0.39 | 0.10 (0.02-0.35) | *0.66* | *0.65* |
| Layered Plaque | 0.21±0.31 | 0.48 (0.19-0.87) | 0.28±0.36 | 0.59 (0.18-0.99) | ***0.029*** | *0.33* |
| Red Thrombus | 0.27±0.36 | 0.19 (0.09-0.38) | 0.40±0.36 | 0.25 (0.03-0.60) | *0.056* | *0.49* |
| White Thrombus | 0.06±0.19 | 1.00 (0.10-1.57) | 0.13±0.27 | 0.88 (0.42-1.01) | *0.52* | *1.00* |
| Plaque Rupture | 0.10±0.26 | 0.17 (0.02-0.40) | 0.25±0.40 | 0.33 (0.01-0.57) | *0.37* | *0.48* |
| Microvessels | 0.18±0.32 | 0.09 (0.04-0.61) | 0.35±0.39 | 0.04 (0.01-0.91) | *0.051* | *0.54* |

Performance reported as mean±SD for inference time and Dice scores, and median (IQR) for HD95. Bold p-values indicate statistical significance (p<0.05).

*HD, Hausdorff distance; IQR, interquartile range; SD, standard deviation.*

### Supplemental Table 3 – Segmentation performance OCT-AID-lite using non-overlapping patient subsets

|  | OCT-AID-lite | |
| --- | --- | --- |
| Class | **Dice** | **TP-Dice** |
| Guidewire | 0.94±0.05 | 0.94±0.05 |
| Catheter | 0.99±0.01 | 0.99±0.01 |
| Lumen | 0.98±0.03 | 0.98±0.03 |
| Sidebranch | 0.48±0.43 | 0.78±0.26 |
| Intima | 0.86±0.11 | 0.86±0.11 |
| Media | 0.78±0.21 | 0.80±0.17 |
| Lipid | 0.62±0.33 | 0.76±0.17 |
| Calcium | 0.55±0.39 | 0.77±0.21 |
| Layered plaque | 0.23±0.33 | 0.55±0.30 |
| Red thrombus | 0.20±0.33 | 0.61±0.28 |
| White thrombus | 0.06±0.18 | 0.35±0.29 |
| Plaque rupture | 0.12±0.30 | 0.83±0.18 |
| Microvessels | 0.21±0.33 | 0.68±0.19 |

Segmentation performance of OCT-AID-lite when trained on non-overlapping patient subsets for manually labeled and pseudo-labeled training data. Performance is reported as mean ± standard deviation for Dice scores across all frames and Dice scores on true-positive (TP) frames.

*TP, true-positive*

### Supplemental Table 4 - Segmentation performance single-fold OCT-AID

|  | Single fold OCT-AID | |
| --- | --- | --- |
| Forward time | 4.81±0.02 | |
| Class | **Dice** | **TP-Dice** |
| Guidewire | 0.94±0.06 | 0.94±0.06 |
| Catheter | 0.99±0.01 | 0.99±0.01 |
| Lumen | 0.98±0.04 | 0.98±0.04 |
| Sidebranch | 0.58±0.42 | 0.81±0.24 |
| Intima | 0.87±0.11 | 0.87±0.11 |
| Media | 0.79±0.21 | 0.81±0.16 |
| Lipid | 0.64±0.33 | 0.77±0.18 |
| Calcium | 0.59±0.39 | 0.79±0.21 |
| Layered plaque | 0.28±0.36 | 0.58±0.31 |
| Red thrombus | 0.40±0.36 | 0.55±0.31 |
| White thrombus | 0.13±0.27 | 0.39±0.35 |
| Plaque rupture | 0.25±0.40 | 0.65±0.38 |
| Microvessels | 0.35±0.39 | 0.74±0.18 |

Segmentation performance of single-fold OCT-AID. Performance is reported as mean ± standard deviation for Dice scores across all frames and Dice scores on true-positive (TP) frames.

*TP, true-positive*

### Supplemental Table 5 – Severity‑stratified calcium arc classification

|  | | Reference annotation | | | |
| --- | --- | --- | --- | --- | --- |
|  |  | No calcium | <90° | 90-180° | >180° |
| Prediction OCT-AID-lite | No calcium | 224 | 14 | 0 | 0 |
|  | <90° | 31 | 72 | 10 | 0 |
|  | 90-180° | 1 | 2 | 16 | 5 |
|  | >180° | 0 | 0 | 1 | 13 |
| Prediction OCT-AID | No calcium | 233 | 17 | 0 | 0 |
|  | <90° | 22 | 70 | 9 | 0 |
|  | 90-180° | 1 | 1 | 17 | 4 |
|  | >180° | 0 | 0 | 1 | 14 |

Severity‑stratified confusion matrices for calcium arc classification, including true‑negative, false‑positive, and false‑negative cases, comparing OCT‑AID‑lite and OCT‑AID predictions with reference annotations.

### Supplemental Table 6 – Classification performance OCT-AID-lite and OCT-AID for all classes

| Class | OCT-AID-lite | | OCT-AID | |
| --- | --- | --- | --- | --- |
|  | **Sensitivity (%)** | **Specificity (%)** | **Sensitivity (%)** | **Specificity (%)** |
| Guidewire | 100.0 (99.0–100.0) | – | 100.0 (99.0–100.0) | – |
| Catheter | 100.0 (99.0–100.0) | – | 100.0 (99.0–100.0) | – |
| Lumen | 100.0 (99.0–100.0) | – | 100.0 (99.0–100.0) | – |
| Sidebranch | 93.2 (84.9–97.0) | 88.0 (83.9–91.1) | 93.2 (84.9–97.0) | 93.4 (90.1–95.6) |
| Intima | 99.7 (98.6–100.0) | – | 100.0 (99.0–100.0) | – |
| Media | 99.5 (98.0–99.9) | 71.4 (50.0–86.2) | 98.4 (96.5–99.3) | 81.0 (60.0–92.3) |
| Lipid | 98.1 (95.3–99.3) | 74.4 (67.5–80.3) | 96.7 (93.4–98.4) | 80.7 (74.2–85.8) |
| Calcium | 89.5 (83.1–93.6) | 87.5 (82.9–91.0) | 87.2 (80.5–91.9) | 91.0 (86.9–93.9) |
| Layered Plaque | 92.6 (85.6–96.4) | 60.9 (55.2–66.3) | 69.5 (59.6–77.8) | 86.7 (82.4–90.1) |
| Red Thrombus | 96.0 (80.5–99.3) | 90.9 (87.5–93.5) | 84.0 (65.3–93.6) | 98.9 (97.2–99.6) |
| White Thrombus | 75.0 (50.5–89.8) | 84.2 (80.1–87.5) | 62.5 (38.6–81.5) | 96.5 (94.1–98.0) |
| Plaque Rupture | 100.0 (72.2–100.0) | 85.0 (81.0–88.2) | 90.0 (59.6–98.2) | 96.6 (94.2–98.0) |
| Microvessels | 86.8 (72.7–94.2) | 77.2 (72.5–81.3) | 84.2 (69.6–92.6) | 91.7 (88.4–94.2) |

Sensitivity and specificity are reported for each class with 95% CI.

*CI, confidence interval.*

### Supplemental Table 7 – Interobserver agreement for observer 1 vs. observer 2

| Class | Interobserver Kappa/ICC^a^ | Total frames | Prevalence | TP frames | Accuracy (%)^b^ | Sensitivity (%)^b^ | Specificity (%)^b^ | PPV (%)^b^ | NPV (%)^b^ |
| --- | --- | --- | --- | --- | --- | --- | --- | --- | --- |
| Lipid plaque | 0.535 (0.486-0.584) | 392 | 132 (33.7%) | 104 (64.2%) | 78.1 (73.7-81.9) | 78.8 (71.1-84.9) | 77.7 (72.2-82.3) | 87.8 (83.0-91.4) | 64.2 (56.6-71.2) |
| Arc | 0.680 (0.529-0.783) | – | – | – | – | – | – | – | – |
| Minimum FCT | 0.564 (0.223-0.739) | – | – | – | – | – | – | – | – |
| Calcium plaque | 0.905 (0.876-0.934) | 392 | 127 (32.4%) | 115 (96.6%) | 95.9 (93.5-97.5) | 90.6 (84.2-94.5) | 98.5 (96.2-99.4) | 96.6 (91.7-98.7) | 95.6 (92.5-97.5) |
| Arc | 0.872 (0.723-0.930) | – | – | – | – | – | – | – | – |
| Depth | 0.829 (0.725-0.890) | – | – | – | – | – | – | – | – |
| Thickness | 0.891 (0.834-0.927) | – | – | – | – | – | – | – | – |

^a^ Cohen’s kappa was used for categorical variables (lipid plaque, calcium plaque), whereas the ICC was used for continuous variables (lipid arc, minimum FCT, calcium arc, calcium depth, and calcium thickness).

^b^ Sensitivity, specificity, PPV, NPV, and accuracy were calculated using observer 1 as the reference rater, whereas κ and ICC quantify agreement between observers without assuming a reference standard.

Performance of observer 1 vs. observer 2 on external validation frames for lipid and calcium plaques. Metrics are reported as value (95% CI).

*CI, confidence interval; FCT, fibrous cap thickness; ICC, intraclass correlation coefficient; NPV, negative predictive value; PPV, positive predictive value; TP, true positive*

### Supplemental Table 8 – Interobserver agreement for observer 2 vs. observer 1

| Class | Interobserver Kappa/ICC^a^ | Total frames | Prevalence | TP frames | Accuracy (%)^b^ | Sensitivity (%)^b^ | Specificity (%)^b^ | PPV (%)^b^ | NPV (%)^b^ |
| --- | --- | --- | --- | --- | --- | --- | --- | --- | --- |
| Lipid plaque | 0.535 (0.486-0.584) | 392 | 162 (41.3%) | 104 (78.8%) | 78.1 (73.7-81.9) | 64.2 (56.6-71.2) | 87.8 (83.0-91.4) | 78.8 (71.1-84.9) | 77.7 (72.2-82.3) |
| Arc | 0.680 (0.529-0.783) | – | – | – | – | – | – | – | – |
| Minimum FCT | 0.564 (0.223-0.739) | – | – | – | – | – | – | – | – |
| Calcium plaque | 0.905 (0.876-0.934) | 392 | 119 (30.4%) | 115 (90.6%) | 95.9 (93.5-97.5) | 96.6 (91.7-98.7) | 95.6 (92.5-97.5) | 90.6 (84.2-94.5) | 98.5 (96.2-99.4) |
| Arc | 0.872 (0.723-0.930) | – | – | – | – | – | – | – | – |
| Depth | 0.829 (0.725-0.890) | – | – | – | – | – | – | – | – |
| Thickness | 0.891 (0.834-0.927) | – | – | – | – | – | – | – | – |

^a^ Cohen’s kappa was used for categorical variables (lipid plaque, calcium plaque), whereas the ICC was used for continuous variables (lipid arc, minimum FCT, calcium arc, calcium depth, and calcium thickness).

^b^ Sensitivity, specificity, PPV, NPV, and accuracy were calculated using observer 2 as the reference rater, whereas κ and ICC quantify agreement between observers without assuming a reference standard.

Performance of observer 1 vs. observer 2 on external validation frames for lipid and calcium plaques. Metrics are reported as value (95% CI).

*CI, confidence interval; FCT, fibrous cap thickness; ICC, intraclass correlation coefficient; NPV, negative predictive value; PPV, positive predictive value; TP, true positive*

### Supplemental Table 9 – Interobserver agreement for observer 1 vs. OCT-AID-lite

| Class | Interobserver Kappa/ICC^a^ | Total frames | Prevalence | TP frames | Accuracy (%) | Sensitivity (%) | Specificity (%) | PPV (%) | NPV (%) |
| --- | --- | --- | --- | --- | --- | --- | --- | --- | --- |
| Lipid plaque | 0.421 (0.372-0.469) | 392 | 132 (33.7%) | 106 (54.9%) | 71.2 (66.5-75.4) | 80.3 (72.7-86.2) | 66.5 (60.6-72.0) | 54.9 (47.9-61.8) | 66.5 (60.6-72.0) |
| Arc | 0.705 (0.546-0.805) | – | – | – | – | – | – | – | – |
| Minimum FCT | 0.585 (0.269-0.750) | – | – | – | – | – | – | – | – |
| Calcium plaque | 0.765 (0.723-0.807) | 392 | 127 (32.4%) | 110 (82.1%) | 89.5 (86.1-92.2) | 86.6 (79.6-91.5) | 90.9 (86.9-93.8) | 82.1 (74.7-87.7) | 93.4 (89.7-95.8) |
| Arc | 0.819 (0.479-0.915) | – | – | – | – | – | – | – | – |
| Depth | 0.846 (0.774-0.895) | – | – | – | – | – | – | – | – |
| Thickness | 0.693 (0.518-0.800) | – | – | – | – | – | – | – | – |

^a^ Cohen’s kappa was used for categorical variables (lipid plaque, calcium plaque), whereas the ICC was used for continuous variables (lipid arc, minimum FCT, calcium arc, calcium depth, and calcium thickness).

Performance of OCT-AID-lite on external validation frames for lipid and calcium plaques, compared with observer 1. Metrics are reported as value (95% CI).

*CI, confidence interval; FCT, fibrous cap thickness; ICC, intraclass correlation coefficient; NPV, negative predictive value; PPV, positive predictive value; TP, true positive*

### Supplemental Table 10 – Interobserver agreement for observer 2 vs. OCT-AID-lite

| Class | Interobserver Kappa/ICCᵃ | Total frames | Prevalence | TP frames | Accuracy (%) | Sensitivity (%) | Specificity (%) | PPV (%) | NPV (%) |
| --- | --- | --- | --- | --- | --- | --- | --- | --- | --- |
| Lipid plaque | 0.698 (0.653-0.744) | 392 | 162 (41.3%) | 148 (76.7%) | 84.9 (81.1-88.1) | 91.4 (86.0-94.8) | 80.4 (74.8-85.0) | 76.7 (70.2-82.1) | 93.0 (88.5-95.8) |
| Arc | 0.750 (0.601-0.836) | – | – | – | – | – | – | – | – |
| Minimum FCT | 0.591 (0.435-0.704) | – | – | – | – | – | – | – | – |
| Calcium plaque | 0.808 (0.769-0.847) | 392 | 119 (30.4%) | 110 (82.1%) | 91.6 (88.4-93.9) | 92.4 (86.2-96.0) | 91.2 (87.3-94.0) | 82.1 (74.7-87.7) | 96.5 (93.5-98.2) |
| Arc | 0.938 (0.896-0.961) | – | – | – | – | – | – | – | – |
| Depth | 0.865 (0.804-0.908) | – | – | – | – | – | – | – | – |
| Thickness | 0.619 (0.446-0.738) | – | – | – | – | – | – | – | – |

^a^ Cohen’s kappa was used for categorical variables (lipid plaque, calcium plaque), whereas the ICC was used for continuous variables (lipid arc, minimum FCT, calcium arc, calcium depth, and calcium thickness).

Performance of OCT-AID-lite on external validation frames for lipid and calcium plaques, compared with observer 2. Metrics are reported as value (95% CI).

*CI, confidence interval; FCT, fibrous cap thickness; ICC, intraclass correlation coefficient; NPV, negative predictive value; PPV, positive predictive value; TP, true positive*

### Supplemental Table 11 – External validation performance of OCT-AID

| Class | Interobserver Kappa/ICCᵃ | Frames with  consensusᵇ | Prevalence | TP frames | Accuracy (%) | Sensitivity (%) | Specificity (%) | PPV (%) | NPV (%) | External testing Kappa/ICCᵃ |
| --- | --- | --- | --- | --- | --- | --- | --- | --- | --- | --- |
| Lipid plaque | 0.535  (0.451–0.619) | 306 | 104 (34.0%) | 93 (89.4%) | 89.5 (85.6-92.5) | 89.4 (82.0-94.0) | 89.6 (84.6-93.1) | 81.6 (73.5-87.6) | 94.3 (90.0-96.8) | 0.772 (0.725-0.819) |
| Arc | 0.516  (0.359–0.644) | – | – | – | – | – | – | – | – | 0.861 (0.744-0.919) |
| Minimum FCT | 0.392  (0.126–0.586) | – | – | – | – | – | – | – | – | 0.796 (0.686-0.867) |
| Calcium plaque | 0.905  (0.860–0.950) | 376 | 115 (30.6%) | 106 (92.2%) | 94.1 (91.3-96.1) | 92.2 (85.8-95.8) | 95.0 (91.7-97.1) | 89.1 (82.2-93.5) | 96.5 (93.5-98.1) | 0.864 (0.829-0.898) |
| Arc | 0.772  (0.566–0.869) | – | – | – | – | – | – | – | – | 0.935 (0.820-0.968) |
| Depth | 0.708  (0.569–0.802) | – | – | – | – | – | – | – | – | 0.949 (0.925-0.966) |
| Thickness | 0.803  (0.715–0.864) | – | – | – | – | – | – | – | – | 0.720 (0.589-0.810) |

^a^ Cohen’s kappa was used for categorical variables (lipid plaque, calcium plaque), whereas the ICC was used for continuous variables (lipid arc, minimum FCT, calcium arc, calcium depth, and calcium thickness).

^b^ Frames for which the independent observers agreed on the presence or absence of the evaluated feature.

Performance of OCT-AID on external validation frames for lipid and calcium plaques, compared with consensus reference. Metrics are reported as value (95% CI).

*CI, confidence interval; FCT, fibrous cap thickness; ICC, intraclass correlation coefficient; NPV, negative predictive value; PPV, positive predictive value; TP, true positive*

### Supplemental Table 12 - Ablation study of quality‑control components and loss functions for lipid and calcium segmentation

| Model | Artifact control | Uncertainty control | $\mathcal{L}_{\text{h}}$ | $\mathcal{L}_{\text{s}}$ | Lipid | | Calcium | |
| --- | --- | --- | --- | --- | --- | --- | --- | --- |
|  |  |  |  |  | **Dice** | **p-value** | **Dice** | **p-value** |
| Ablation 1 | - | - | $\mathcal{L}_{\text{DF}}^{(\text{M})}$ | - | 0.71±0.17 | ***<0.01*** | 0.66±0.26 | ***<0.01*** |
| Ablation 2 | - | - | $\mathcal{L}_{\text{DF}}^{(\text{M})}+\mathcal{L}_{\text{DF}}^{(\text{P})}$ | - | 0.76±0.17 | 0.84 | 0.78±0.20 | 0.72 |
| Ablation 3 | - | - | $\mathcal{L}_{\text{DF}}^{(\text{M})}$ | MSE | 0.61±0.19 | ***<0.01*** | 0.47±0.29 | ***<0.01*** |
| Ablation 4 | - | - | $\mathcal{L}_{\text{DF}}^{(\text{M})}$ | KL | 0.75±0.17 | ***0.022*** | 0.69±0.28 | ***<0.01*** |
| Ablation 5 | - | - | $\mathcal{L}_{\text{DF}}^{(\text{M})}+\mathcal{L}_{\text{DF}}^{(\text{P})}$ | MSE | 0.63±0.19 | ***<0.01*** | 0.55±0.27 | ***<0.01*** |
| Ablation 6 | - | - | $\mathcal{L}_{\text{DF}}^{(\text{M})}+\mathcal{L}_{\text{DF}}^{(\text{P})}$ | KL | 0.75±0.17 | 0.90 | 0.75±0.23 | 0.98 |
| Ablation 7 | Yes | - | $\mathcal{L}_{\text{DF}}^{(\text{M})}+\mathcal{L}_{\text{DF}}^{(\text{P})}$ | KL | 0.74±0.18 | 0.16 | 0.74±0.23 | 0.84 |
| Ablation 8 | - | Yes | $\mathcal{L}_{\text{DF}}^{(\text{M})}+\mathcal{L}_{\text{DF}}^{(\text{P})}$ | KL | **0.76**±**0.16** | 0.13 | **0.77**±**0.23** | 0.86 |
| OCT-AID-lite | Yes | Yes | $\mathcal{L}_{\text{DF}}^{(\text{M})}+\mathcal{L}_{\text{DF}}^{(\text{P})}$ | KL | **0.76**±**0.16** | - | **0.77**±**0.22** | - |

Dice scores are reported as mean ± SD on true‑positive frames. P‑values indicate statistical comparisons between each ablation experiment and OCT-AID‑lite. Bold p-values indicate statistical significance (*p<0.05*).

*DF, Dice–Focal; KL, Kullback–Leibler;* $\mathcal{L}_{\text{h}}$*, hard loss;* $\mathcal{L}_{\text{s}}$*, soft loss; M, manual labeled data; MSE, mean squared error; P, pseudo-labeled data; SD, standard deviation.*

### Supplemental Table 13 - True‑positive Dice scores across ablation experiments and the final OCT‑AID‑lite model

| Class | Ablation 1 | Ablation 2 | Ablation 3 | Ablation 4 | Ablation 5 | Ablation 6 | Ablation 7 | Ablation 8 | OCT-AID-lite |
| --- | --- | --- | --- | --- | --- | --- | --- | --- | --- |
| Guidewire | 0.93±0.06 | 0.94±0.05 | 0.86±0.13 | 0.93±0.05 | 0.91±0.07 | 0.94±0.05 | 0.94±0.04 | 0.94±0.04 | 0.94±0.04 |
| Catheter | 0.99±0.01 | 0.99±0.01 | 0.99±0.01 | 0.99±0.01 | 0.99±0.01 | 0.99±0.01 | 0.99±0.01 | 0.99±0.01 | 0.99±0.01 |
| Lumen | 0.98±0.04 | 0.98±0.03 | 0.96±0.07 | 0.98±0.03 | 0.97±0.06 | 0.98±0.03 | 0.98±0.04 | 0.98±0.03 | 0.98±0.03 |
| Sidebranch | 0.70±0.29 | 0.78±0.25 | 0.56±0.27 | 0.76±0.25 | 0.58±0.31 | 0.76±0.26 | 0.75±0.27 | 0.79±0.24 | 0.78±0.25 |
| Intima | 0.83±0.12 | 0.86±0.11 | 0.78±0.14 | 0.85±0.11 | 0.80±0.14 | 0.86±0.10 | 0.85±0.11 | 0.86±0.11 | 0.86±0.11 |
| Media | 0.74±0.19 | 0.81±0.15 | 0.64±0.20 | 0.77±0.17 | 0.68±0.20 | 0.80±0.15 | 0.79±0.17 | 0.79±0.16 | 0.79±0.16 |
| Lipid | 0.71±0.17 | 0.76±0.17 | 0.61±0.19 | 0.75±0.17 | 0.63±0.19 | 0.75±0.17 | 0.74±0.18 | 0.76±0.16 | 0.76±0.16 |
| Calcium | 0.66±0.26 | 0.78±0.20 | 0.47±0.29 | 0.69±0.28 | 0.55±0.27 | 0.75±0.23 | 0.74±0.23 | 0.77±0.23 | 0.77±0.22 |
| Layered Plaque | 0.39±0.27 | 0.55±0.26 | 0.19±0.22 | 0.40±0.27 | 0.21±0.22 | 0.54±0.28 | 0.58±0.27 | 0.51±0.30 | 0.50±0.29 |
| Red Thrombus | 0.54±0.27 | -^a^ | -^a^ | 0.61±0.23 | -^a^ | 0.65±0.25 | 0.55±0.33 | 0.56±0.31 | 0.65±0.24 |
| White Thrombus | 0.38±0.31 | -^a^ | -^a^ | 0.39±0.32 | -^a^ | 0.52±0.28 | 0.41±0.29 | 0.36±0.32 | 0.39±0.31 |
| Plaque Rupture | 0.55±0.32 | -^a^ | -^a^ | 0.37±0.30 | -^a^ | 0.57±0.38 | 0.60±0.35 | 0.61±0.37 | 0.67±0.30 |
| Microvessels | 0.58±0.22 | -^a^ | -^a^ | 0.64±0.21 | -^a^ | 0.64±0.25 | 0.63±0.23 | 0.70±0.19 | 0.66±0.21 |

^a^ Dice score not defined because the model did not predict any instances of this class.

Performance reported as mean ± SD for true-positive Dice scores.

Ablation settings: Ablation 1–5: no QC. Ablation 1: manual hard loss only. Ablation 2: manual and pseudo hard loss. Ablation 3: manual hard + MSE soft loss. Ablation 4: manual hard + KL soft loss. Ablation 5: manual and pseudo hard + MSE soft loss. Ablation 6: manual and pseudo hard + KL soft loss. Ablation 7: artifact-based QC + manual and pseudo hard + KL soft loss. Ablation 8: uncertainty-based QC + manual and pseudo hard + KL soft loss.

*KL, Kullback–Leibler; MSE, mean squared error; QC, quality control; SD, standard deviation*

### Supplemental Table 14 - Dice scores across ablation experiments and the final OCT‑AID‑lite model

| Class | Ablation 1 | Ablation 2 | Ablation 3 | Ablation 4 | Ablation 5 | Ablation 6 | Ablation 7 | Ablation 8 | OCT-AID-lite |
| --- | --- | --- | --- | --- | --- | --- | --- | --- | --- |
| Guidewire | 0.93±0.06 | 0.94±0.05 | 0.86±0.13 | 0.93±0.05 | 0.91±0.07 | 0.94±0.05 | 0.94±0.04 | 0.94±0.04 | 0.94±0.04 |
| Catheter | 0.99±0.01 | 0.99±0.01 | 0.99±0.01 | 0.99±0.01 | 0.99±0.01 | 0.99±0.01 | 0.99±0.01 | 0.99±0.01 | 0.99±0.01 |
| Lumen | 0.98±0.04 | 0.98±0.03 | 0.96±0.07 | 0.98±0.04 | 0.97±0.06 | 0.98±0.03 | 0.98±0.04 | 0.98±0.03 | 0.98±0.03 |
| Sidebranch | 0.40±0.41 | 0.50±0.42 | 0.22±0.32 | 0.47±0.42 | 0.20±0.33 | 0.52±0.42 | 0.43±0.43 | 0.51±0.43 | 0.48±0.43 |
| Intima | 0.83±0.13 | 0.86±0.11 | 0.78±0.14 | 0.85±0.11 | 0.80±0.14 | 0.86±0.10 | 0.85±0.11 | 0.86±0.11 | 0.86±0.12 |
| Media | 0.72±0.23 | 0.78±0.20 | 0.61±0.24 | 0.76±0.20 | 0.64±0.25 | 0.78±0.19 | 0.77±0.20 | 0.77±0.20 | 0.77±0.20 |
| Lipid | 0.56±0.33 | 0.61±0.33 | 0.44±0.32 | 0.59±0.34 | 0.45±0.33 | 0.60±0.34 | 0.58±0.35 | 0.61±0.34 | 0.62±0.33 |
| Calcium | 0.42±0.38 | 0.53±0.40 | 0.23±0.31 | 0.50±0.39 | 0.28±0.34 | 0.52±0.40 | 0.48±0.40 | 0.57±0.39 | 0.55±0.39 |
| Layered Plaque | 0.13±0.24 | 0.22±0.32 | 0.05±0.14 | 0.13±0.25 | 0.05±0.14 | 0.21±0.31 | 0.21±0.32 | 0.21±0.32 | 0.21±0.31 |
| Red Thrombus | 0.20±0.31 | -^a^ | -^a^ | 0.22±0.32 | -^a^ | 0.26±0.36 | 0.20±0.33 | 0.25±0.35 | 0.27±0.36 |
| White Thrombus | 0.07±0.19 | -^a^ | -^a^ | 0.06±0.19 | -^a^ | 0.07±0.21 | 0.04±0.16 | 0.06±0.19 | 0.06±0.19 |
| Plaque Rupture | 0.05±0.18 | -^a^ | -^a^ | 0.04±0.15 | -^a^ | 0.08±0.24 | 0.07±0.23 | 0.11±0.29 | 0.10±0.27 |
| Microvessels | 0.16±0.29 | -^a^ | -^a^ | 0.17±0.30 | -^a^ | 0.16±0.31 | 0.14±0.28 | 0.19±0.33 | 0.18±0.32 |

^a^ Dice score not defined because the model did not predict any instances of this class.

Performance reported as mean ± SD for Dice scores.

Ablation settings: Ablation 1–5: no QC. Ablation 1: manual hard loss only. Ablation 2: manual and pseudo hard loss. Ablation 3: manual hard + MSE soft loss. Ablation 4: manual hard + KL soft loss. Ablation 5: manual and pseudo hard + MSE soft loss. Ablation 6: manual and pseudo hard + KL soft loss. Ablation 7: artifact-based QC + manual and pseudo hard + KL soft loss. Ablation 8: uncertainty-based QC + manual and pseudo hard + KL soft loss.

*KL, Kullback–Leibler; MSE, mean squared error; QC, quality control; SD, standard deviation*

### Supplemental Table 15 - Performance comparison on true-positive frames between OCT-AID-lite, DeepLabv3^+^ and SegFormer

|  | OCT-AID-lite | DeepLabv3^+3^ | p-value | SegFormer^4^ | p-value |
| --- | --- | --- | --- | --- | --- |
| Parameters (x 10^6^) | 10.8 | 14.3 | | 13.7 | |
| Forward-pass time (s) | 0.10±0.03 | 0.12±0.03 | ***<0.01*** | 0.13±0.05 | ***<0.01*** |
| Guidewire | 0.94±0.04 | 0.93±0.08 | ***<0.01*** | 0.93±0.07 | ***<0.01*** |
| Catheter | 0.99±0.01 | 0.99±0.01 | ***<0.01*** | 0.99±0.01 | ***<0.01*** |
| Lumen | 0.98±0.03 | 0.98±0.04 | ***<0.01*** | 0.98±0.04 | ***<0.01*** |
| Sidebranch | 0.78±0.25 | 0.79±0.23 | ***<0.01*** | 0.77±0.24 | *0.39* |
| Intima | 0.86±0.11 | 0.84±0.11 | ***<0.01*** | 0.85±0.10 | ***<0.01*** |
| Media | 0.79±0.16 | 0.77±0.16 | ***<0.01*** | 0.77±0.16 | ***<0.01*** |
| Lipid | 0.76±0.16 | 0.74±0.17 | ***<0.01*** | 0.75±0.17 | *0.54* |
| Calcium | 0.77±0.22 | 0.79±0.15 | *0.15* | 0.79±0.18 | *0.73* |
| Layered Plaque | 0.50±0.29 | -^a^ | -^b^ | 0.60±0.26 | ***<0.01*** |
| Red Thrombus | 0.65±0.24 | -^a^ | -^b^ | -^a^ | -^b^ |
| White Thrombus | 0.39±0.31 | -^a^ | -^b^ | -^a^ | -^b^ |
| Plaque Rupture | 0.67±0.30 | -^a^ | -^b^ | 0.65±0.16 | *0.19* |
| Microvessels | 0.66±0.21 | -^a^ | -^b^ | -^a^ | -^b^ |

^a^ Dice score not defined because the model did not predict any instances of this class.
^b^ p-value not obtainable because one or both models did not predict any instances of this class.

Performance of OCT-AID-lite compared with DeepLabv3^+^ and SegFormer. Metrics are reported as mean ± SD for inference time and Dice scores on true-positive frames. Bold p-values indicate statistical significance (*p<0.05*).

*SD, standard deviation.*

### Supplemental Table 16 - Performance comparison between OCT-AID-lite, DeepLabv3^+^ and SegFormer

|  | OCT-AID-lite | DeepLabv3^+^ ^5^ | p-value | SegFormer ^4^ | p-value |
| --- | --- | --- | --- | --- | --- |
| Parameters (x 10^6^) | 10.8 | 14.3 | | 13.7 | |
| Forward-pass time (s) | 0.10±0.03 | 0.12±0.03 | ***<0.01*** | 0.13±0.05 | ***<0.01*** |
| Guidewire | 0.94±0.04 | 0.93±0.08 | ***<0.01*** | 0.93±0.07 | ***<0.01*** |
| Catheter | 0.99±0.01 | 0.99±0.01 | ***<0.01*** | 0.99±0.01 | ***<0.01*** |
| Lumen | 0.98±0.03 | 0.98±0.04 | ***<0.01*** | 0.98±0.04 | ***<0.01*** |
| Sidebranch | 0.48±0.43 | 0.51±0.42 | ***<0.01*** | 0.46±0.42 | 0.63 |
| Intima | 0.86±0.12 | 0.84±0.12 | ***<0.01*** | 0.85±0.11 | ***<0.01*** |
| Media | 0.77±0.20 | 0.74±0.21 | ***<0.01*** | 0.75±0.20 | ***<0.01*** |
| Lipid | 0.62±0.33 | 0.58±0.34 | ***0.016*** | 0.59±0.34 | 0.66 |
| Calcium | 0.55±0.39 | 0.59±0.37 | ***<0.01*** | 0.60±0.37 | ***<0.01*** |
| Layered Plaque | 0.21±0.31 | -^a^ | -^b^ | 0.25±0.34 | ***<0.01*** |
| Red Thrombus | 0.27±0.36 | -^a^ | -^b^ | -^a^ | -^b^ |
| White Thrombus | 0.06±0.19 | -^a^ | -^b^ | -^a^ | -^b^ |
| Plaque Rupture | 0.10±0.26 | -^a^ | -^b^ | 0.20±0.32 | ***<0.01*** |
| Microvessels | 0.18±0.32 | -^a^ | -^b^ | -^a^ | -^b^ |

^a^ Dice score not defined because the model did not predict any instances of this class.

^b^ p-value not obtainable because one or both models did not predict any instances of this class.

Performance of OCT-AID-lite compared with DeepLabv3^+^ and SegFormer. Metrics are reported as mean ± SD for inference time and Dice scores. Bold p-values indicate statistical significance (*p<0.05*).

*SD, standard deviation.*

# References

1.	Zhou Y, Li Z, Bai S, Wang C, Chen X, Han M*, et al.* Prior-Aware Neural Network for Partially-Supervised Multi-Organ Segmentation. *2019 IEEE/CVF International Conference on Computer Vision (ICCV)* 2019:10671-10680. doi:

2.	Noothout JMH, Lessmann N, van Eede MC, van Harten LD, Sogancioglu E, Heslinga FG*, et al.* Knowledge distillation with ensembles of convolutional neural networks for medical image segmentation. *J Med Imaging (Bellingham)* 2022;**9**:052407. doi: 10.1117/1.Jmi.9.5.052407

3.	Chen L-C, Zhu Y, Papandreou G, Schroff F, Adam H. Encoder-Decoder with Atrous Separable Convolution for Semantic Image Segmentation. In: *European Conference on Computer Vision*. *2018*.

4.	Xie E, Wang W, Yu Z, Anandkumar A, Álvarez JM, Luo P. SegFormer: Simple and Efficient Design for Semantic Segmentation with Transformers. *ArXiv* 2021;**abs/2105.15203**. doi:

5.	Chen LC, Papandreou G, Kokkinos I, Murphy K, Yuille AL. DeepLab: Semantic Image Segmentation with Deep Convolutional Nets, Atrous Convolution, and Fully Connected CRFs. *IEEE Trans Pattern Anal Mach Intell* 2018;**40**:834-848. doi: 10.1109/tpami.2017.2699184
